# Interlaboratory study on Sb_2_S_3_ interplay between structure, dielectric function, and amorphous-to-crystalline phase change for photonics

**DOI:** 10.1016/j.isci.2022.104377

**Published:** 2022-05-10

**Authors:** Yael Gutiérrez, Anna P. Ovvyan, Gonzalo Santos, Dilson Juan, Saul A. Rosales, Javier Junquera, Pablo García-Fernández, Stefano Dicorato, Maria M. Giangregorio, Elena Dilonardo, Fabio Palumbo, Mircea Modreanu, Josef Resl, Olga Ishchenko, Guy Garry, Tigers Jonuzi, Marin Georghe, Cornel Cobianu, Kurt Hingerl, Christoph Cobet, Fernando Moreno, Wolfram H.P. Pernice, Maria Losurdo

**Affiliations:** 1 CNR ICMATE, Corso Stati Uniti 4, I-35127, Padova, Italy; 2 Institute of Physics, University of Münster, Heisenbergstraße 11, 48149 Münster, Germany; 3 Departmento de Física Aplicada, Universidad de Cantabria, Avda. Los Castros S/n, 39005 Santander, Spain; 4 Departamento de Ciencias de La Tierra y Física de La Materia Condensada, Universidad de Cantabria, Cantabria Campus Internacional, Avda. de Los Castros S/n, 39005 Santander, Spain; 5 Tyndall National Institute-University College Cork, Lee Maltings, Dyke Parade, Cork T12 R5CP, Ireland; 6 Center for Surface and Nanoanalytics, Johannes Kepler University, 4040 Linz, Austria; 7 TE-OX, 21 Rue Jean Rostand, 91400 Orsay, France; 8 VLC Photonics S.L. Universidad Politécnica de Valencia (access I) Camino de Vera S/n - 46022Valencia, Spain; 9 NANOM MEMS Srl, G. Cosbuc 9, 505400 Rasnov, Brasov, Romania; 10 Heidelberg University, Kirchhoff-Institute for Physics, Im Neuenheimer Feld 227, 69120 Heidelberg, Germany

**Keywords:** Applied sciences, Materials science, Photonics

## Abstract

Antimony sulfide, Sb_2_S_3_, is interesting as the phase-change material for applications requiring high transmission from the visible to telecom wavelengths, with its band gap tunable from 2.2 to 1.6 eV, depending on the amorphous and crystalline phase. Here we present results from an interlaboratory study on the interplay between the structural change and resulting optical contrast during the amorphous-to-crystalline transformation triggered both thermally and optically. By statistical analysis of Raman and ellipsometric spectroscopic data, we have identified two regimes of crystallization, namely 250°C ≤ *T* < 300°C, resulting in *Type-I* spherulitic crystallization yielding an optical contrast Δ*n* ∼ 0.4, and 300 ≤ *T* < 350°C, yielding *Type-II* crystallization bended spherulitic structure with different dielectric function and optical contrast Δ*n* ∼ 0.2 below 1.5 eV. Based on our findings, applications of on-chip reconfigurable nanophotonic phase modulators and of a reconfigurable high-refractive-index core/phase-change shell nanoantenna are designed and proposed.

## Highlights

• Study of the dielectric function of amorphous and crystalline Sb2S3 phase change material
• Identification of two crystallization regimes for the phase change of Sb2S3
• Design of Sb2S3 on-chip reconfigurable photonic phase modulator
• Design of reconfigurable high-refractive index core/phase-change shell nanoantenna

## Graphical abstract

**Figure:**
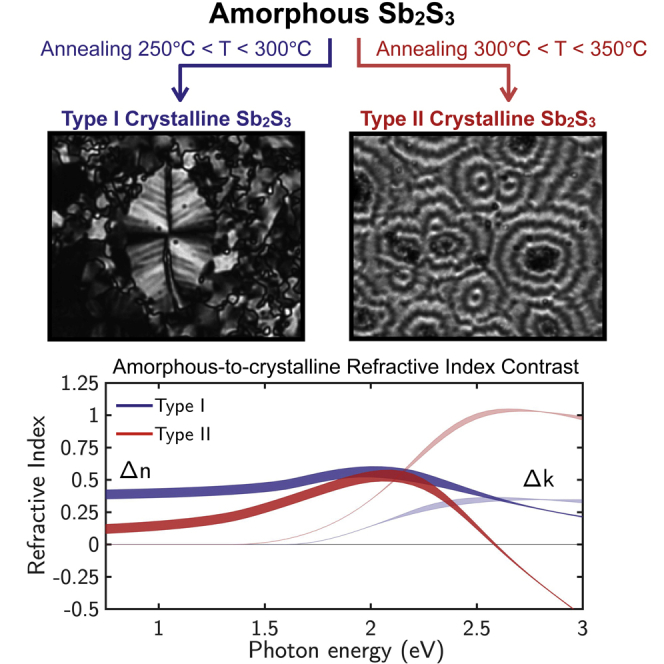

## Introduction

Phase-change materials (PCMs) that can be switched between amorphous and crystalline phases by thermal annealing or laser irradiation are being exploited in a wide range of reconfigurable photonic platforms that expand from programmable photonics (Stegmaier et al., 2017; Delaney et al., 2021), neuromorphic computing (Feldmann et al., 2019), non-volatile and rewritable data storage (Ríos et al., 2015; Wuttig and Yamada, 2007), to tunable metasurfaces and flat optics with amplitude/phase control (Abdollahramezani et al., 2020), cloaking (Lepeshov et al., 2019), and reflective displays (Carrillo et al., 2019). Most PCMs contain chalcogenides elements such as tellurium (Te) and/or pnictogens such as antimony (Sb), like the prototypical Ge_2_Sb_2_Te_5_ (GST) and its alloys (Wuttig and Yamada, 2007). GST is established as a mature technology because of its large optical contrast (Δ*n* = 2.17 and Δ*k* = 0.785 at λ = 1,550 nm) (Pernice and Bhaskaran, 2012) and resistance difference (Δρ > 1,000 Ω⋅m at λ = 1,550 nm) between the amorphous and crystalline phases, fast phase switching (nanosecond scale), large number of cycles of reversible transitions (billions of reproducible cycles), and relatively low transition temperature (crystallization temperature *T*_c_ ≈ 170°C and melting temperature of *T*_m_ ≈ 600°C) (Wuttig and Yamada, 2007). Nevertheless, GST has a non-null extinction coefficient of the crystalline (*k* = 1.49) and amorphous phases (*k* = 0.12) at telecommunication wavelengths, introducing absorption losses that impose limitations to applications in phase modulation schemes where phase control independent of amplitude changes of the propagating signal is required.

In general, as the chalcogenide element increases in atomic number, i.e., sulfur → selenium → tellurium, the bandgap of the PCM tends to decrease. Therefore, Te-based PCMs have rather small gaps, typically below 1 eV, making difficult to realize reconfigurable photonic components at visible frequencies.

Thus, in the last decade, numerous attempts have focused on identifying PCMs with large band gap and a pronounced optical contrast at photon energies higher than 1 eV to enable optical amplitude and phase modulations schemes in the visible range (Müller et al., 2021).

In the quest for low-loss and high-bandgap PCMs, the sesqui-chalcogenide antimony sulfide, Sb_2_S_3_, with octahedral-like atomic arrangement and a ratio of glassy and melting temperatures *T*_g_ (220°C)/*T*_m_ (540°C) ≈ 0.5 (Wuttig and Yamada, 2007) is gaining increasing interest (Dong et al., 2019; Delaney et al., 2020) also because it has been shown to exhibit topological state transition under pressure (Sorb et al., 2016). Sb_2_S_3_ has been reported to have an optical band gap of 2.05 and 1.72 eV for the amorphous and crystalline phase, respectively, and a refractive index contrast Δ*n* ≈ 0.6 with negligible losses as the extinction coefficient, *k*, is less than 10^−5^ in both amorphous and crystalline phases at 1,550 nm (Delaney et al., 2020; Dong et al., 2019). Interestingly, the phase transition temperature of Sb_2_S_3_ is accessible by diode laser irradiation as Sb_2_S_3_ has a crystallization activation energy of 2.0 eV (Sreekanth et al., 2019), which is similar to that of Ge_2_Sb_2_Te_5_ (2.3 eV) (Guo et al., 2019). Therefore, crystallization is achieved by thermal annealing Sb_2_S_3_ to temperatures around 250°C, whereas amorphization involves heating above its *T*_m_ of 540°C and quenching to “freeze-in” the disordered state.

Sb_2_S_3_ synthesis over large area has been reported using a wide range of methods including chemical bath deposition (Kondrotas et al., 2018; Cobianu et al., 2021), electrophoretic deposition (Hassam et al., 2021), atomic layer deposition (Kim et al., 2014) and RF sputtering (Gao et al., 2019). In the 1990s, Sb_2_S_3_ was widely studied for write-once-read-many times (WORM) optical storage applications, as problems related to sulfur loss upon phase change leading to film degradation were reported (Arun and Vedeshwar, 1996, 1997). Very recently, Dong et al. (2019) reported the laser amorphous-crystalline-amorphous switching and questioned the WORM classification, although they did not measur the cycle endurance of Sb_2_S_3_. Delaney et al. (2020) showed a decay in reflection change from 5% to 1% over 2,000 cycles of phase-change. On the other hand, very recently, Gao et al. (2021) demonstrated that multi-pulse laser irradiation with low pulse energy can improve cycling durability with respect to single-pulse, high-energy irradiation. Despite further studies on the cycles endurance and reversibility of Sb_2_S_3_ are needed, integration of Sb_2_S_3_ in photonic circuits is already being exploited in the design of Mach–Zehnder interferometers (MZIs) operating in the C and O communication bands (Faneca et al., 2021).

In order to exploit the potential of Sb_2_S_3_ in those applications, it is highly desirable to understand better the changes of structural property and to control the optical contrast that can be realized.

Considering the technological relevance that Sb_2_S_3_ is gaining, this work presents results of an interlaboratory study aimed at understanding how the structure and bonding nature of Sb_2_S_3_ determines its electronic and optical properties and how those properties change upon the amorphous-to-crystalline transformation triggered by thermal annealing and laser irradiation. In the Round Robin test, Raman spectroscopy was considered for establishing statistically structural fingerprints, as it is a sensitive probe for isostructural transitions (Efthimiopoulos et al., 2016), whereas spectroscopic ellipsometry was exploited to obtain the spectral dependence of the refractive index, *n*, and extinction coefficient, *k*, in the broad range of 1,700–190 nm (0.75–6.50 eV), in order to evaluate the spectral applicability of Sb_2_S_3_ in reconfigurable devices. Those analyses were further corroborated by extensive chemical (energy dispersive X-ray spectroscopy – EDX; X-ray photoelectron spectroscopy – XPS), morphological (atomic force microcopy – AFM; scanning electron microcopy – SEM) and optical (polarimetry) characterization. Therefore, first, the crystalline c-Sb_2_S_3_ and amorphous, a-Sb_2_S_3_, properties are statistically analyzed in order to control and validate the interplay between phase transformation and optical contrast during thermal and laser crystallization. Specifically, independently of the annealing atmosphere and of film thickness, and depending on temperature we have identified two regimes of annealing, namely 250 ≤ *T* < 300°C resulting in *Type-I* crystallized spherulitic structural network with an optical contrast Δ*n* ≈ 0.4, and 300 ≤ *T* < 350°C, resulting in *Type-II* crystallized bended structural network with different dielectric function and optical contrast Δ*n* ≈ 0.2 at telecom wavelengths. Then, based on the established optical properties, applications of an on-chip reconfigurable nanophotonic phase modulator and of a reconfigurable high-refractive-index (HRI) core/PCM-shell (Si/Sb_2_S_3_) nanoantenna are designed and proposed.

## Results

### Single crystal Sb_2_S_3_ (*c* Sb_2_S_3_): definition of band structure, dielectric function, and Raman spectrum

This paragraph aims at defining the reference single crystal, c-Sb_2_S_3_, structure, band structure, and corresponding anisotropic dielectric function.

**Figure 1:**
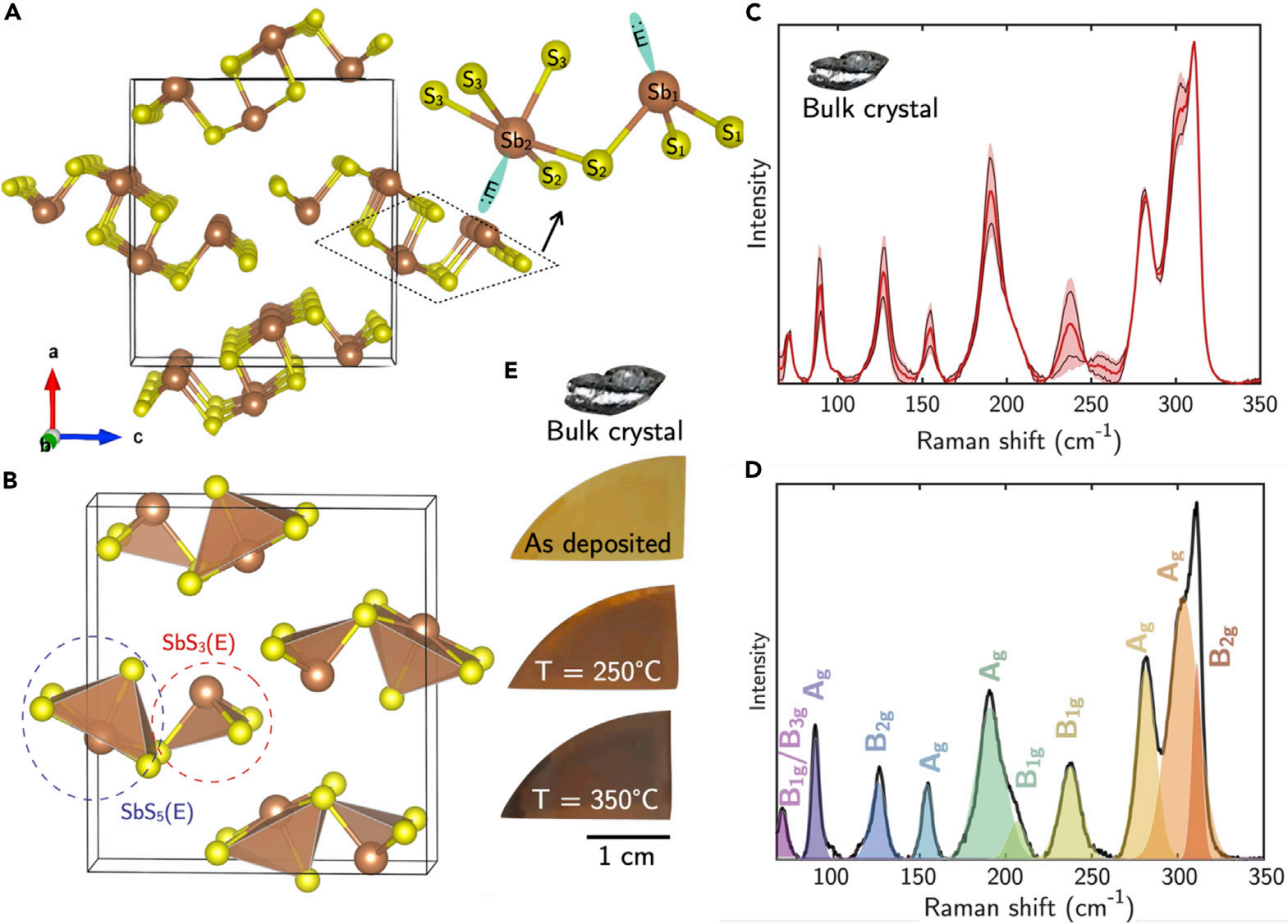
Interplay between structure and Raman spectrum of crystalline Sb_2_S_3_ (A) Sb_2_S_3_ crystalline structure. Arrangement of the nearest sulfur S(1), S(2), and S(3) atoms for Sb in the two different sites of Sb(1) and Sb(2), forming (B) SbS_3_(E) and SbS_5_(E) units; (E) indicates the Sb electrons lone-pairs. (C) Statistical analysis of the Raman spectra of single crystal Sb_2_S_3_; the shadow indicates the statistical variation of the relative amplitude of the modes. The red line spectrum represents the mean value. (D) Raman spectra of single crystal Sb_2_S_3_ indicating fitting of the Raman modes with Gaussian line shape function. (E) Picture of the bulk crystal and of ∼150-nm-thick Sb_2_S_3_ samples as deposited (amorphous) and after annealing at different temperatures (crystallized) for 5 min.

As shown in Figure 1A, c-Sb_2_S_3_ is a biaxial anisotropic material that crystallizes in an orthorhombic structure of space group symmetry *Pnma* (no. 62) with lattice parameters *a* = 1.13107 nm, *b* = 0.3863 nm, and *c* = 1.12285 nm (Bayliss and Nowacki, 1972). This *Pnma* phase resembles a layered structure consisting of (Sb_4_S_6_)_n_ ribbon-like chains along the short *b*-axis held together by longer weaker bonds with significant (but not entirely) van der Waals character, as they involve the electrons lone-pair in the 5s^2^orbital of Sb-atoms. The coordination number of both Sb- and S-atoms is not straightforward, as can also be inferred from Figures 1A and 1B, as three different S-sites and two different Sb-sites can be identified, with the Sb(1) coordinated to three S-atoms in SbS_3_(E), and the Sb(2) coordinated to five S-atoms in SbS_5_(E) units, being (E) the electrons lone-pair of Sb. Furthermore, those SbS_3_(E) and SbS_5_(E) units also have different kinds of bonds, namely short and strong intra-ribbon bonds and long and weaker inter-ribbon bonds; specifically, Sb(2) in SbS_5_(E) forms intra-ribbon bonds with the five S-atoms and two more inter-ribbon bonds with most distant S-atoms (McKenna, 2021). Those weak bonds as well as the Sb electrons lone-pair introduce Peierls-like distortion (Gaspard, 2016), giving flexibility in reconfiguring the Sb_2_S_3_ structure as it will be discussed below.

Because of those various types of Sb- and S-sites and bonds, the Raman spectrum of c-Sb_2_S_3_ is very complex, and various spectra are reported in literature, also depending on the excitation laser source. Figure 1C shows the statistical distribution of the relative amplitude of the unpolarized Raman modes of c-Sb_2_S_3_ resulting from interlaboratory measurements. According to the group theory, 30 Raman-active modes are expected (Liu et al., 2014), i.e., Γ = 10 A_g_ + 5B_1g_ + 10B_2g_ + 5B_3g_. As shown in Figure 1D, in the range 50–350 cm^−1^, we can resolve 10 Raman bands assigned according to (Liu et al., 2014), i.e., the modes B_1g_/B_3g_ at 70.4 cm^−1^, A_g_ at 89.7 cm^−1^ and B_2g_ at 125.9 cm^−1^ (Ibáñez et al., 2016), A_g_ at 154.5 cm^−1^ and the modes A_g_ at 191.1 cm^−1^, B_1g_ at 206.6 cm^−1^, B_1g_ at 237.7 cm^−1^, A_g_ at 281.4 cm^−1^, A_g_ at 303.3 cm^−1^, and B_2g_ at 311.2 cm^−1^. The 191 and 238 cm^−1^ bands represent the anti-symmetric and symmetric S-Sb-S bending modes, respectively. Although the 281.4 and 303.3 cm^−1^ anti-symmetric and symmetric S-Sb-S stretching modes are usually ascribed to the trigonal pyramidal vibration modes of the SbS_3_(E) units (Benjamin et al., 2015), their link with neighboring SbS_3_(E) and SbS_5_(E) units makes the unique assignment of pure symmetric and anti-symmetric stretching modes of SbS_3_ and SbS_5_ units difficult (Ibáñez et al., 2016). Noteworthy, the unit cell in Figure 1B shows that c-Sb_2_S_3_ has 50% of SbS_3_(E) units and 50% of SbS_5_(E) units. From the mean value spectrum resulting from the statistical analysis, we identify the following modes ratio as representative of *c*-Sb_2_S_3_, namely the A_g_(303cm^−1^)/A_g_(281cm^−1^) ≈ 1.5 and the A_g_(303cm^−1^)/A_g_(191cm^−1^) ≈ 1.5. Considering that the A_g_(303cm^−1^) clearly has more contributions, we make the hypothesis that the A_g_(303cm^−1^) is more representative of SbS_5_(E) units, whereas the A_g_(281cm^−1^) is more representative of the SbS_3_(E) units; this hypothesis will be further supported during the discussion in the next paragraphs on structural modifications during the amorphous-to-crystalline transformation.

**Figure 2:**
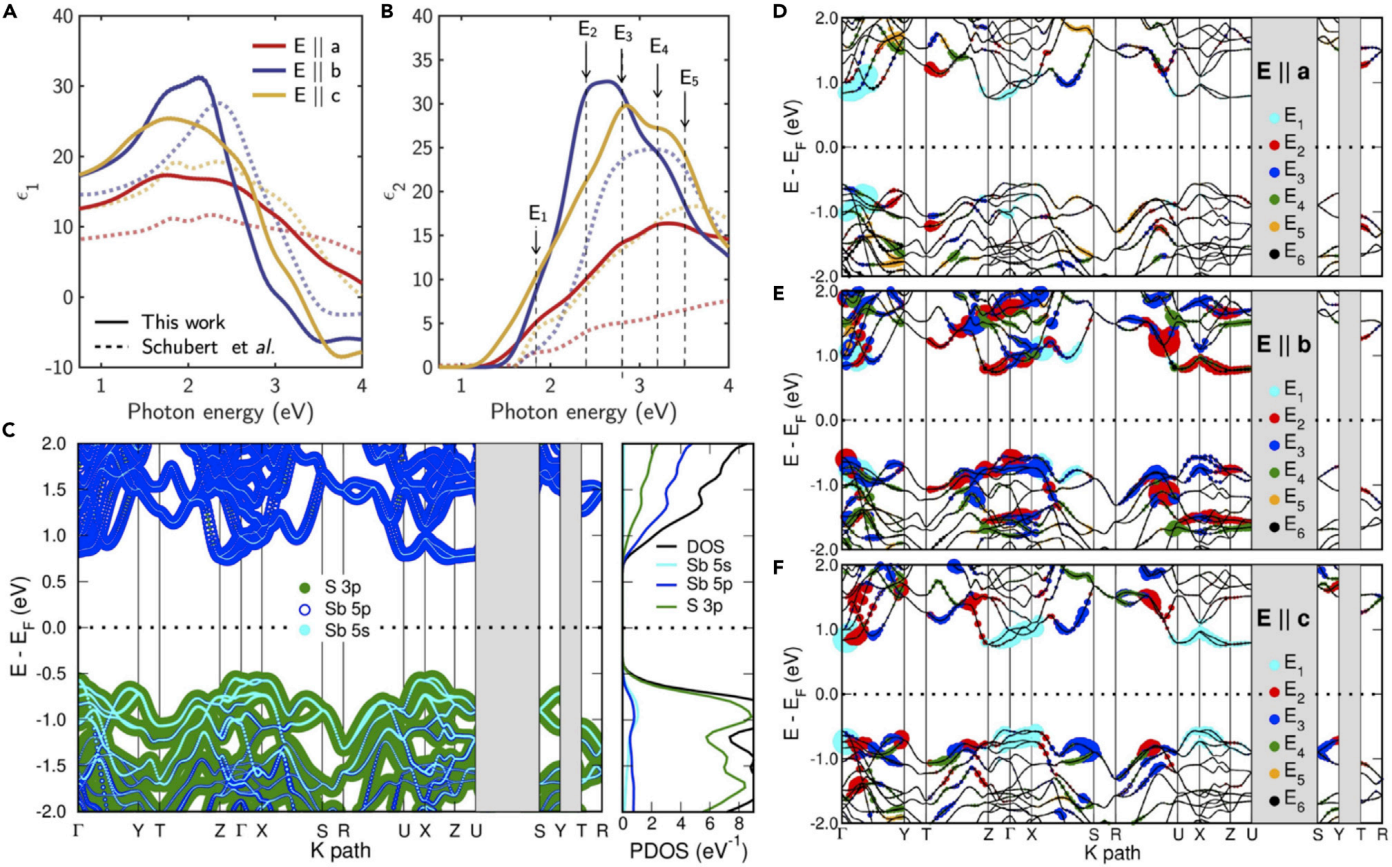
Interplay between the calculated dielectric function, band structure, and DOS of Sb_2_S_3_ Calculated (A) real, $\in_{1}$, and (B) imaginary, $\in_{2}$, parts of dielectric functions of c-Sb_2_S_3_ for polarizations along the crystallographic axes *a*, *b*, and *c* as compared with values reported by Schubert et al. (2004). (C) Orbital-projected band diagram and density of states of Sb_2_S_3_ on the S 3*p*, Sb 5*p*, and Sb 5*s* shells. The size of the marker is proportional to the strength of the contribution. Band diagram of Sb_2_S_3_ representing the bands contributing to the interband transitions responsible of the CPs in the dielectric function for polarizations along the crystallographic (D) *a-axis*, (E) *b-axis*, and (F) *c-axis* (the size of the symbol is a measure of the probability amplitude). The table at the bottom summarizes the energy of the main critical points also compared with literature data from Schubert et al. (2004) and Shutov et al. (1969)).

Similarly, scattered data about the crystalline Sb_2_S_3_ dielectric function are present in literature, as often referred to a crystalline state without clarifying if referring to a single crystal or a polycrystalline material. Interestingly, optical constants were measured at specific wavelengths many years ago on natural cleavage surfaces by Tyndall and Drude (Drude, 1888; Tyndall, 1923). More recently, Shubert et al. (Schubert et al., 2004) reported the complex dielectric function tensor of Sb_2_S_3_ obtained by cutting and measuring by generalized spectroscopic ellipsometry the Sb_2_S_3_ crystal along the (100) (a-plane), (010) (b-plane), and (001) (c-plane). Six different interband transitions associated with critical points (CPs) in the dielectric function were identified, in good agreement with those measured by Shutov et al. (1969) by polarized reflectivity. Nevertheless, no information about the bands involved in the interband transitions was provided. In order to establish a correlation between the dielectric function and interband transitions based on the band structure and the dipolar transition matrix elements between occupied and unoccupied single-electron eigenstates, the complex dielectric function has been calculated for c-Sb_2_S_3_ using first-order time-dependent perturbation theory as implemented in SIESTA (Soler et al., 2002). Figures 2A and 2B show the real, $\in_{1}$, and imaginary, $\in_{2}$, part of the complex dielectric function, $\in =\in_{1}+i\in_{2}$, calculated for c-Sb_2_S_3_ for polarization parallel to the crystallographic axes (E||*a*, E||*b*, E||*c*). Noteworthy, the calculated dielectric function is in good agreement with those reported by Schubert et al. (2004). A critical points analysis by using the minimum of the second derivative spectra of the dielectric function, d^2^ $\in_{1}$/dE^2^ and d^2^ $\in_{2}$/dE^2^ (not shown here) calculated numerically reveals CPs in agreement with the experimental measurements by Schubert (Schubert et al., 2004), as shown in the table at the bottom of Figure 2. For the assignment of the CPs, Figure 2C shows the orbital-projected bands (fatbands) and the projected density of states (PDOS) of bulk Sb_2_S_3_ representing the contribution of the S 3*p*, Sb 5*s*, and Sb 5*p* orbitals to each band. We notice that the highest valence bands are comprised primarily of S orbitals with 3*p* character and a minor contribution of a mixing between Sb 5*s* and Sb 5*p* levels and the featured structure of the PDOS at around −1 eV has being suggested to indicate the formation of electron lone-pairs on the Sb-ions by Carey et al. (2014). The valence band maximum (VBM) is slightly displaced from the Γ-point in the high symmetry line ΓZ (this direction corresponds to the c-axis of the (Sb_4_S_6_)_n_ chains perpendicular to the zigzag arrangement). Likely, the conduction band minimum (CBM) is located at the same high symmetry line but closer to the Z-point. This results in an indirect fundamental band gap of 1.30 eV and a higher direct gap of 1.33eV. Our results are in agreement with previously reported first principles calculations using PBE functional (Sun et al., 2008; Carey et al., 2014), although the location of the VBM and CBM in the Brillouin zone does not have total consensus throughout the related bibliography. The shape of the band structure resembles the anisotropy of the crystal structure of Sb_2_S_3_, having flatter bands in directions along *a-* and *c*-axes and larger band widths perpendicular to both.

Considering the different contributions of the Sb and S atomic orbitals as in Figures 2D–2F), the following interband transitions can be assigned to the CPs, as in the table. The peak E_1_ involves direct transitions near the Γ-point (in the ΓY direction) for all polarization directions between the highest valence bands and lower conduction bands comprising mostly occupied S3p and Sb5s orbitals and empty Sb5p. The peak labeled E_2_ presents contributions spread over the momentum space, weaker for E||*a* case with respect to perpendicular polarizations. Direct transitions occur between valence bands at −0.9 eV and higher conduction bands around 1.5 eV and also between the lower valence bands at −1.5 eV and conduction bands in the minimum valleys. The peak E_3_ is also composed of transitions delocalized in k-space. The contributions of E||*b* and E||*c* are larger with respect to E||*a* polarization; for the first case, the stronger transition probabilities arise close to Γ, Z and X-points. The peak E_4_ comprise vertical excitations from low lying valence bands (<−1 eV) and unoccupied conduction bands above 1.5eV mostly, also showing a wide distribution over k-space. Peaks E_5_ and E_6_ present relative lower transition probabilities with respect to the formers;, it is noticeable that intense contributions to E_5_ are found for E||*a* polarization in ΓY and XS directions.

### Amorphous Sb_2_S_3_ films (a-Sb_2_S_3_): interlaboratory study of the structural (Raman) and optical (ellipsometry) properties

This paragraph aims at defining and validating the structural and optical fingerprints of a-Sb_2_S_3,_ namely the dielectric function and the corresponding Raman spectrum. The XPS analysis revealed that the analyzed a-Sb_2_S_3_ films were almost stoichiometric with an S/Sb atomic ratio of 1.45 ± 0.05 estimated by both XPS and EDX analyses.

**Figure 3:**
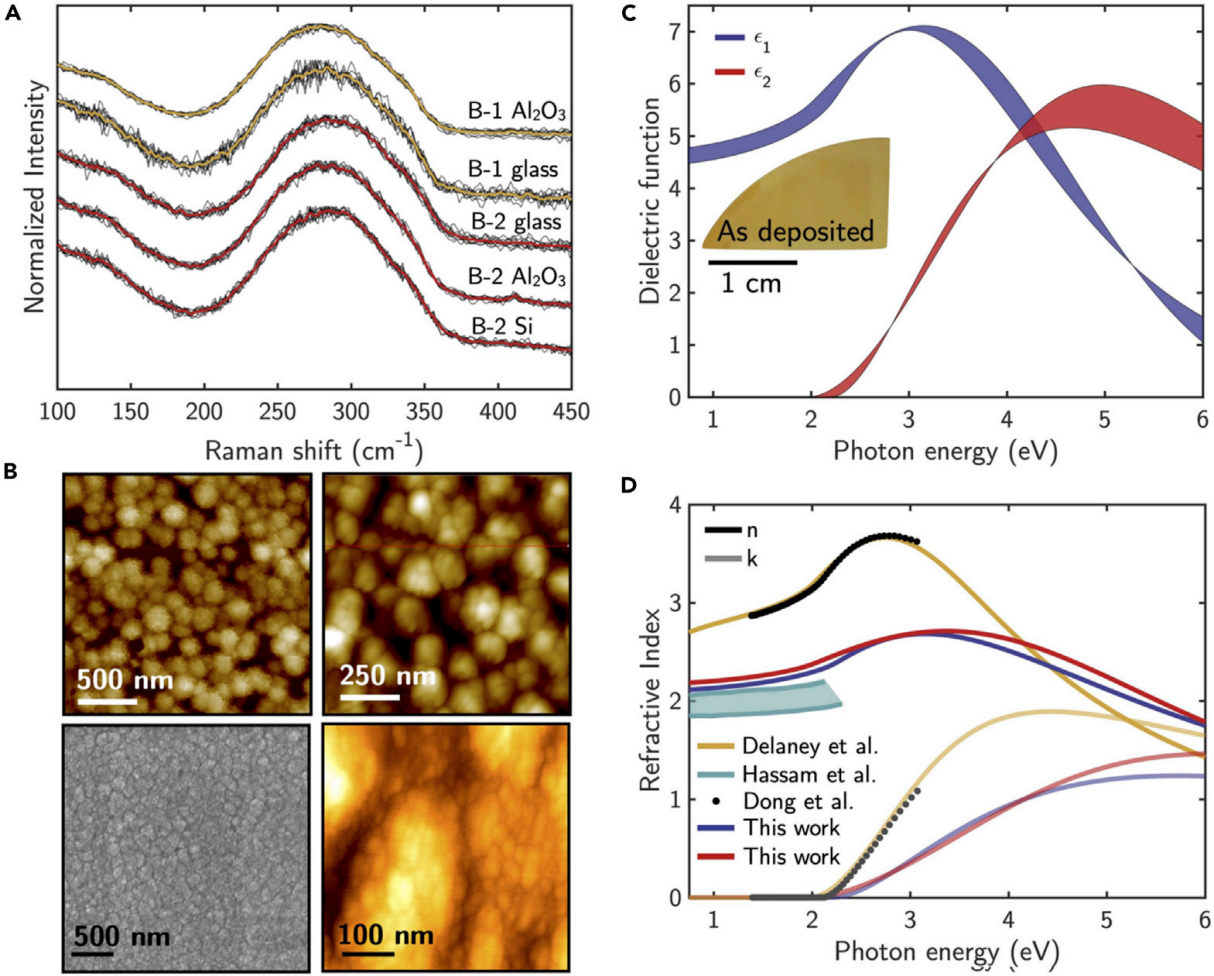
Statistical analysis of Raman spectra, optical properties, and morphology characterizing amorphous Sb_2_S_3_ Statistical analysis of Raman spectra of a-Sb_2_S_3_ films measured from two different batches (B-1 and B-2) on glass, sapphire and Si(100). Individual measurements are plotted in black, whereas the colored line represents the mean value. (B) Topographical images recorded by SEM (gray) and AFM (colored) in various laboratories for a-Sb_2_S_3_ films. (C) Statistical variation of the energy-dependent behavior of the real, ε_1_, and imaginary, ε_2_, parts of the a-Sb_2_S_3_ dielectric function. (D) Refractive index, *n*, and extinction coefficient, *k*, of a-Sb_2_S_3_ also compared with the data in literature (Delaney et al., 2020; Dong et al., 2019; Hassam et al., 2021).

Figure 3A shows the statistical analysis of Raman spectra of a-Sb_2_S_3_ films from two different batches (Batch #1 (B-1) and Batch #2 (B-2)) produced 6 months apart on various substrates of glass, sapphire, and Si(100) measured in different laboratories with different instruments. The Raman spectra of a-Sb_2_S_3_ show good reproducibility and is characterized by a broad and intense mode at ≈ 285 cm^−1^, which could originate from the mixture of stretching and bending modes of distorted SbS_3_(E) and SbS_5_(E) units and, hence, from the envelope of broadened A_g_ at 281.4 cm^−1^, B_1g_ at 303.3 cm^−1^, and A_g_ at 311.2 cm^−1^ vibrational modes. Indeed, a-Sb_2_S_3_ has larger Peierls-like distortion (Gaspard, 2016) than c-Sb_2_S_3_, with bonding angles deviated slightly from 90°. Specifically, it has been reported that chalcogen-centered S-bonds slightly shift to larger angles (94.11°), whereas the corresponding Sb-centered angles decrease to 88.81° (Xu et al., 2021). Interestingly, such structural distortions are responsible for the wider band gap of a-Sb_2_S_3_ than c-Sb_2_S_3_ and for the different electronic and optical properties, as measured by ellipsometry.

Figure 3C shows the statistical variation of the energy-dependent behavior of the real, *ε*_*1*_, and imaginary, *ε*_*2*_, parts of the isotropic a-Sb_2_S_3_ dielectric function, resulting in a bandgap of 2.2 ± 0.1 eV; all analyzed a-Sb_2_S_3_ samples behave in a similar way, regardless the thickness; the shadowed area indicates the effect of the local Peierls-like distortions on the dielectric function. The isotropy of the a-Sb_2_S_3_ dielectric function was proven by imaging polarimetry, as the Mueller Matrix measured on different a-Sb_2_S_3_ films revealed the identity matrix, typical of isotropic materials, as shown in Figure S1 (Gutierrez et al., 2022).

The a-Sb_2_S_3_ index of refraction (*n*) and extinction coefficient (*k*) are compared with literature data in Figure 3D. The different amplitude of the curves can be explained by the different contribution of voids resulting from a large number of Sb-atoms electrons lone-pair (Xu et al., 2021), depending on the stoichiometry of the deposited amorphous samples. Furthermore, more voids mean larger Peierls-like distortions, therefore, emphasizing the role of those distortions on the a-Sb_2_S_3_ dielectric function. Specifically, the strength of an optical transition and, hence, the maximum of *ε*_*2*_*,* decreases with increasing Peierls distortion, i.e., shortening Sb-S bonds. Therefore, tailoring Peierls-like distortion in the a-Sb_2_S_3_ could be a way to increase the optical contrast between the amorphous and crystalline phases.

The surface topographical images recorded by SEM and AFM in various laboratories for a-Sb_2_S_3_ films with thicknesses of ≈150 nm, shown in Figure 3B, confirms the presence of voids, with films characterized by a grainy-like morphology. The measured grain size and roughness (root-mean-square roughness, RMS) are statistically as 30 ± 5 and −3.5 ± 0.5 nm, respectively. The irregular shapes of the grains stem from the fact that at room temperature during deposition, the kinetic energy is not enough to induce the coalescence of the grains (Tigau, 2007).

### Sb_2_S_3_ amorphous-to-crystalline phase change by thermal annealing and laser crystallization

Here we address the interplay between structure and optical properties induced by both thermal and laser crystallization.

**Figure 4:**
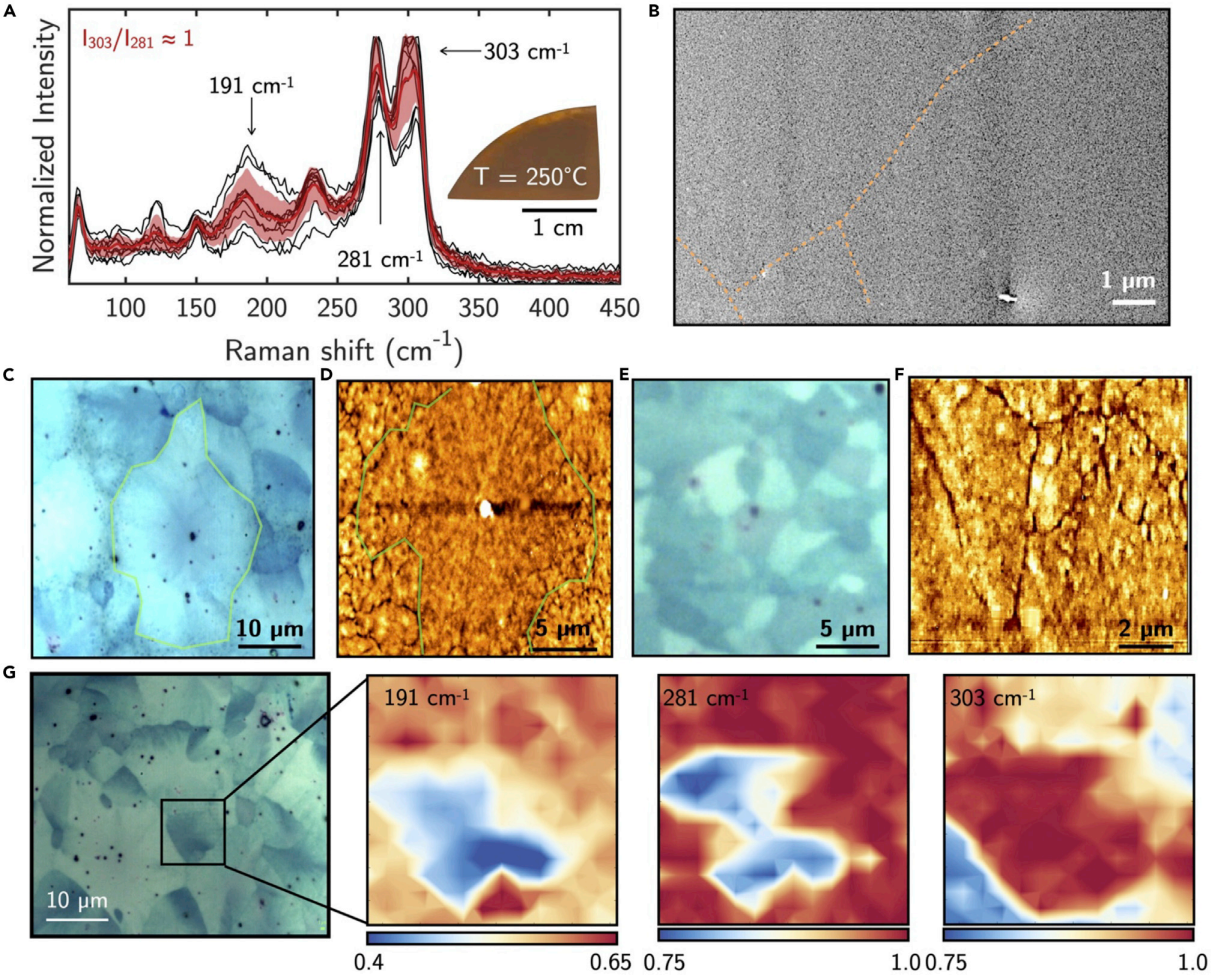
Structural and morphological characteristics of *Type-I* Sb_2_S_3_ crystallized at 250°C ≤ *T* < 300°C (A) Statistical distribution of the Raman spectra of thermally crystallized Sb_2_S_3_ films at 250°C ≤ *T* < 300°C. Individual measurements are plotted in black, whereas the red spectrum and shadowed regions represent the mean value and standard deviation, respectively. (B) SEM image showing the flat morphology of *Type-I* crystallized c-Sb_2_S_3_. The orange dashed line is to guide eye to grain boundaries visible in the SEM image as well as in the AFM images. (C and E) Optical micrographs and (D and F) AFM topography images of a spherulitic crystal and of randomly distributed crystallites (crystallized at *T* = 250°C for 5 min). (G) Raman maps of the 191, 281, and 303 cm^−1^ modes taken at a region containing several randomly oriented larger crystallites (crystallized at *T* = 250°C for 10 min).

Figure 4 shows the representative optical, SEM, and AFM images of crystallized Sb_2_S_3_ upon thermal annealing. The annealing was run under various conditions in different laboratories (see STAR Methods). Noteworthy, data from the various laboratories could be grouped in two different kinds of structures/morphologies independently of the annealing in vacuum, argon, and air, revealing the role mainly of the annealing temperature (*T* = 250–300°C and 300–350°C) on the evolution of structure upon thermal crystallization. Specifically, *Type-I* crystallized c-Sb_2_S_3_, with an almost unchanged S/Sb ratio of 1.40 ± 0.05 as from XPS analysis, consists of spherulitic crystals as in Figures 4C and 4D (Shtukenberg et al., 2012; Crist and Schultz, 2016) surrounded by randomly oriented smaller crystallites as in Figure 4E. The spherulites consist of dense fibrils radially grown from a single nucleation point, as can also be seen in the AFM image of Figure 4D. This spherulitic thermal crystallization of a-Sb_2_S_3_ films is consistent with previous work (Bolotov et al., 1970; Sokol et al., 1986; Gutierrez et al., 2022). Those spherulitic faceted crystallites obtained in the range 250–300°C increase in the size and uniformity of distribution as well as compactness with the increase in annealing temperature, as shown in Figures 4C (*T* = 300°C) and 4E (*T* = 250°C). The grain boundaries and surface features are clearly seen in the AFM and SEM images in Figures 4B–4F, with large grains of the order of tens of micrometers. Interestingly, *Type-I* c-Sb_2_S_3_is characterized by a very low surface roughness (RMS = 1.9 ± 0.3 nm).

**Figure 5:**
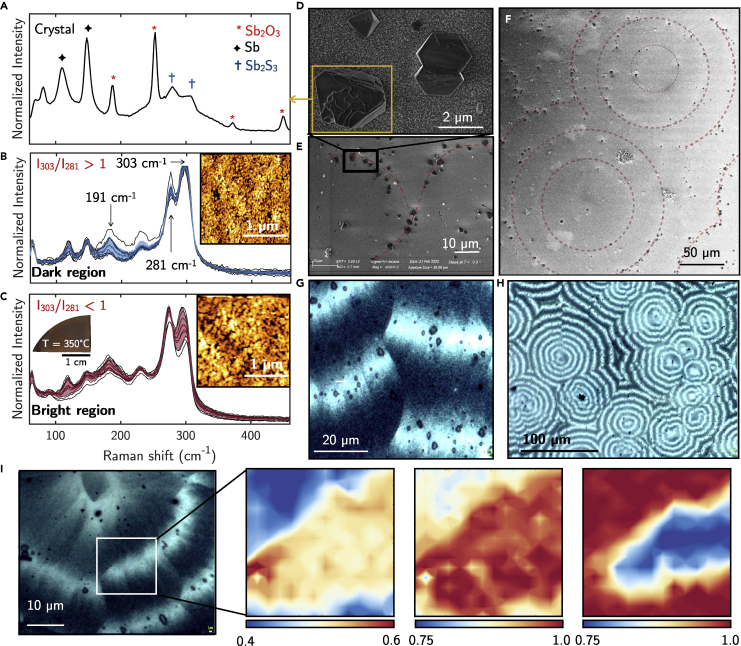
Structural and morphological characteristics of *Type-II* Sb_2_S_3_ crystallized at *T* > 300°C Raman and morphological features of *Type-II* crystallized c-Sb_2_S_3_. (A) Raman spectrum of the segregated crystals of Sb and Sb_2_O_3_ appearing on the surface of bright bands. (B) Statistical analysis of the Raman spectra on the dark and (C) bright bands of the bended spherulites. Individual measurements are plotted in black, whereas the colored line and shadowed regions represent the mean value and standard deviation, respectively. (D–F) SEM images of the surface of *Type-II* c-Sb_2_S_3_. (G and H) Optical micrographs of the *Type-II* c-Sb_2_S_3_- showing the bended spherulites. (I) Raman maps of the 191, 281, and 303 cm^−1^ modes taken at bright and dark bands. AFM morphologies of the dark and bright bands are shown as inset in (B) and (C), respectively.

The Raman spectrum of *Type-I* crystallized Sb_2_S_3_ was statistically studied as shown in Figure 4A. Large amplitude variations characterize the modes at 191, 281, and 303 cm^−1^. Interestingly, the statistically analysis of the intensity ratio between modes at 303 and 281 cm^−1^ has an average value A_g_(303cm^−1^)/A_g_(281cm^−1^) ≈ 1. The role of the crystallites orientations on the Raman amplitude is further emphasized in Figure 4G, where the Raman maps of the modes at 191, 281, and 303 cm^−1^ taken in a region of randomly oriented crystallites exposing different faces show a different amplitude of the modes. The large spherulitic crystals as those considered in the map of Figures 5G–5I are characterized by the A_g_(303cm^−1^)/A_g_(281cm^−1^) ≈ 1.5 similarly to the c-Sb_2_S_3._

*Type-II* crystallized Sb_2_S_3,_ which resulted in a changed S/Sb ratio of 0.95 ± 0.05 from both XPS and EDX analyses, shows banded patterns spherulites (see Figure S4), characterized by periodic rings with a width of approximately 10 μm as shown in Figures 5G–5I. Careful SEM and optical inspections as in Figures 5D–5G show hexagonal and octahedral Sb and Sb_2_O_3_ (see Raman analysis Figure 5A) crystals localized on the white bright bands. Statistical Raman analysis on the different bright and dark bands shows significant differences in the intensity of the Raman modes at 191, 281, and 303 cm^−1^ in those bands. Specifically, Raman spectra at the dark bands are characterized by an intensity ratio A_g_(303cm^−1^)/A_g_(281cm^−1^) >1. Conversely, Raman spectra at the bright bands are characterized by A_g_(303cm^−1^)/A_g_(281cm^−1^) < 1 with an increased intensity of the mode at 191 cm^−1^. This is further shown in Figure 5I by the Raman maps of the modes at 191, 281, and 303 cm^−1^ taken in an area comprising both dark and bright bands. These maps clearly show a higher intensity of the modes at 191 and 281 cm^−1^ and lower intensity of the 303 cm^−1^ mode at the bright bands. This behavior is inverted for the dark bands. The AFM of *Type-II* crystallized Sb_2_S_3_ also shows different morphologies and roughness of the bright (RMS = 5.1 nm) and dark bands (RMS = 4.0 nm), which is higher than the RMS of *Type-I*. Thus, from this contrast of bands, we infer the mobility of Sb atoms along different crystallographic directions. On this, more detailed study is in progress to identify them.

A further insight into the structural and optical properties of the *Type-I* and *Type-II* c-Sb_2_S_3_ was obtained by imaging polarimetry of the thermally crystallized Sb_2_S_3_ films. A differential analysis of the Mueller matrix (MM) was performed using the Mueller Matrix Differential Decomposition (Arteaga and Kahr, 2013), from which the linear and circular birefringence and dichroism of the crystallized film can be obtained. Specifically, the dichroic properties are owing to the anisotropy of extinction coefficient Δ*k*_*p,q*_ for two orthogonal polarization states (*p,q*). The birefringent properties stem from the anisotropy in the real refractive index Δ*n*_*p,q*_, entailing different phase shifts for two orthogonal polarization states (Gil Pérez and Ossikovski, 2017). Therefore, expressions for linear dichroism in the *x*–*y* axes, linear dichroism in the 45–135°and circular dichroism ($LD_{xy}$, $LD_{45{}^{\circ}}$ and CD), and linear birefringence in the *x*–*y* and 45–135° axes and circular birefringence ($LB_{xy}$, $LB_{45{}^{\circ}}$ and CB) can be written as

(Equation 1) $$\begin{array}{cc} LD_{xy}=\frac{2\pi }{\lambda }\text{Δ}k_{x-y} & LB_{xy}=\frac{2\pi }{\lambda }\text{Δ}n_{x-y}\\ LD_{45{}^{\circ}}=\frac{2\pi }{\lambda }\text{Δ}k_{45{}^{\circ}-135{}^{\circ}} & LB_{45{}^{\circ}}=\frac{2\pi }{\lambda }\text{Δ}n_{45{}^{\circ}-135{}^{\circ}}\\ CD=\frac{2\pi }{\lambda }\text{Δ}k_{L-R} & CB=\frac{2\pi }{\lambda }\text{Δ}n_{L-R} \end{array}$$

**Figure 6:**
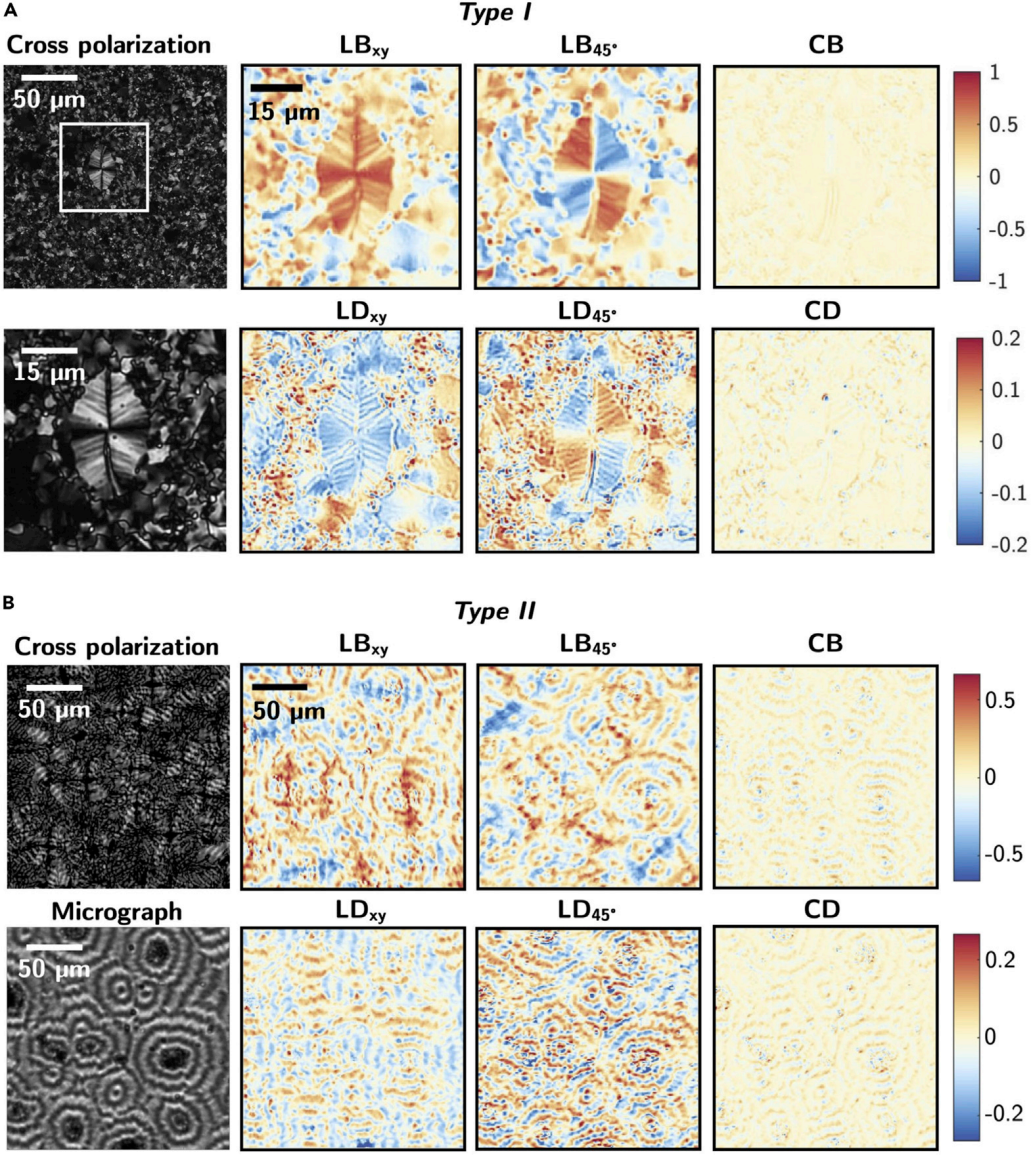
Polarimetric and Mueller Matrix Differential Decomposition of *Type-I* and *Type-II* thermally crystallized Sb_2_S_3_ (A) *Type-I* and (B) *Type-II* thermally crystallized Sb_2_S_3_ cross polarization, micrographs, and Mueller Matrix Differential Decomposition. $LD_{xy}$, $LD_{45{}^{\circ}}$, and CD parameters are the linear dichroism in the *x*–*y* axes, linear dichroism in the 45°–135° and circular dichroism, respectively, whereas $LB_{xy}$, $LB_{45{}^{\circ}}$, and CB parameters are linear birefringence in the *x*–*y* and 45–135° axes and circular birefringence. The larger spherulitic crystal highlighted by the white square is equivalent to the crystal shown in previous Figures 4C and D by optical micrography and AFM.

Mueller matrices obtained for an excitation wavelength of 633 nm for the annealed *a*-Sb_2_S_3_ films in the range of 250–350°C are shown in Figures S1–S3. Unlike in the case of the MM of a-Sb_2_S_3_ in Figure S1, for *Type I* and *Type II* c-Sb_2_S_3_, strong signals are observed in the non-diagonal elements of the MM, especially on the elements *m*_*24*_, *m*_*42*_, *m*_*34*_ and *m*_*43*_ as seen in Figures S2 and S3. As shown in Figure 6, through the MMDD, the $LD_{xy}$, $LD_{45{}^{\circ}}$, CD, $LB_{xy}$, $LB_{45{}^{\circ}}$, and CB parameters were extracted from the MMs.

In the case of *Type I* in Figure 6A, these parameters show a strong birefringent behavior (LB ≠ 0) with a lower LD contribution arising from the different refractive index and extinction coefficients associated with the dielectric tensor of Sb_2_S_3_ (Schubert et al., 2004). Both CD and CB are negligible. Cross-polarization micrographs reveal big crystalline domains showing a slightly distorted maltase cross-pattern (i.e., the four dark perpendicular cones diverging from the center of the crystal) typical of spherulites (as those in Figure 4C) (Bolotov et al., 1970; Gutierrez et al., 2022; Sokol et al., 1986). Therefore, *Type-I* c-Sb_2_S_3_ has a polycrystalline texture consisting of randomly oriented micron-size crystallites surrounding larger spherulitic crystalline domains.

For *Type II,* polarimetric measurements in Figure 6B support a crystallization process forming banded spherulitic crystals, as confirmed by the banded Maltese cross pattern seen in the cross-polarization. The MMDD for *Type-II* shows strong signals of linear birefringent and dichroism taking alternating signs between consecutives bands. Similar behavior, although with lower signals, can be seen in both CD and CB. Recent studies by Cui et al. (2014) concluded that concentric bands of CB of alternating sign is a universal signature of helical twisting in banded spherulites.

Both *Type-I* and *Type-II* c-Sb_2_S_3_ show local birefringent and dichroic behavior within the microscopic crystalline domains. Nevertheless, at the macroscopic level, considering the radial symmetry of spherulites as well as the randomly oriented microcrystallites, they result in an overall isotropic response for the crystallized film, supporting the isotropic dielectric function determined by ellipsometry.

**Figure 7:**
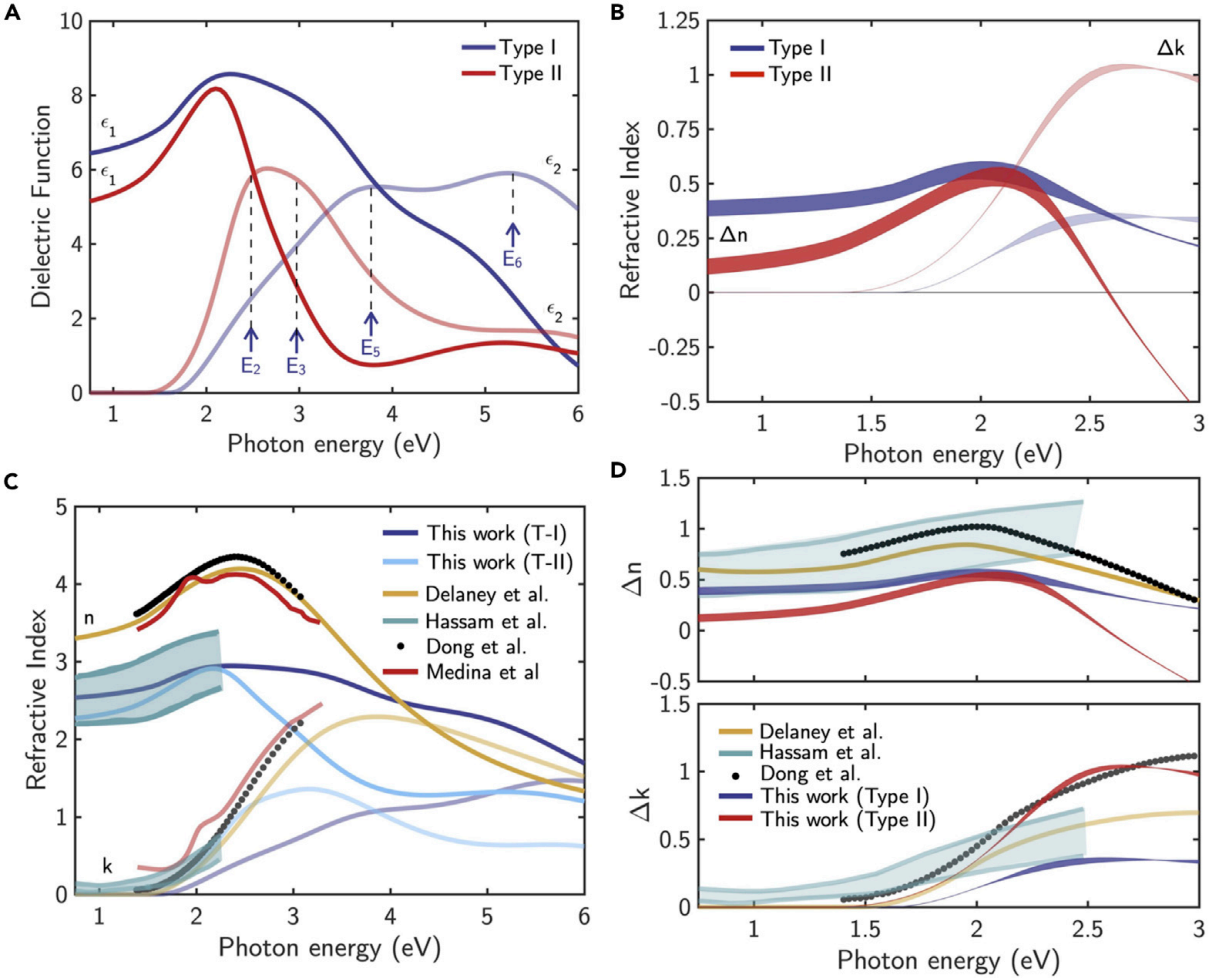
Dielectric function and optical contrast for *Type-I* and *Type-II* crystallized c-Sb_2_S_3_ (A) Real, ε_1_, and imaginary, ε_2_, part of the dielectric function for *Type-I* and *Type-II* crystallized c-Sb_2_S_3_. (B) Refractive index contrast between amorphous and *Type-I* and *Type-II* crystallized Sb_2_S_3_. (C) Refractive index *n*, and extinction coefficient, *k*, for *Type-I* and *Type-II* c-Sb_2_S_3_ compared with values from literature (Delaney et al., 2020; Dong et al., 2019; Hassam et al., 2021). (D) Refractive index contrast between amorphous and *Type-I* and *Type-II* c-Sb_2_S_3_ also compared with values from literature (Delaney et al., 2020; Dong et al., 2019; Hassam et al., 2021).

Those two types of crystallization and structures can induce a different optical contrast during the amorphous-to-crystalline transformation. This is confirmed by the different optical properties reported in Figure 7. Figure 7A shows the real, ε_1_, and imaginary, ε_2_, part of the dielectric function of *Type-I* and *Type-II* c-Sb_2_S_3_ as determined from the ellipsometric analysis. Interestingly, ε_2_ of spherulitic *Type-I* c-Sb_2_S_3_ shows broadened CPs and a shape of ε_2_ similar to that along the *a*-axis (see Figure 2), leading to an optical contrast of Δ*n* ∼ 0.4. Consistent with the different structure highlighted in Figures 5 and 6, a different dielectric function spectrum characterizes *Type-II* c-Sb_2_S_3_. By comparing those spectra with c-Sb_2_S_3_ in Figure 2, it can be inferred that *Type-II* crystallized samples have a dielectric function resembling the average of the three components along the three axis of the dielectric tensor in Figure 2 (consistently with the bending scheme in Figure S4), leading to an optical contrast during the amorphous-to-crystalline transition of Δ*n* ∼ 0.2 in the spectral region <1.5 eV. The spectra of the refractive index, *n*, and extinction coefficient, *k*, are compared with data from literature in Figures 7C and 7D, whereas the values of absorption edge are compared with literature data in Table 1.

**Table 1:** Values energy band gap and absorption edge for crystallized and amorphous Sb_2_S_3_ films

Ref.	Crystalline–band gap (eV)	Amorphous–absorption edge (eV)
This work	1.61 ± 0.05	2.20 ± 0.05
(Dong et al., 2019)	1.72	2.05
(Hassam et al., 2021)	1.27–1.47	2.09–2.20
(Tigau, 2007)	1.71	1.96
(Medina-Montes et al., 2016)	1.63–1.70	2.00–2.03
(Medina-Montes et al., 2017)	1.65–1.68	1.96–2.06
(Cui et al., 2021)	1.73	–
(Sotelo Marquina et al., 2017)	1.7	2.2

**Figure 8:**
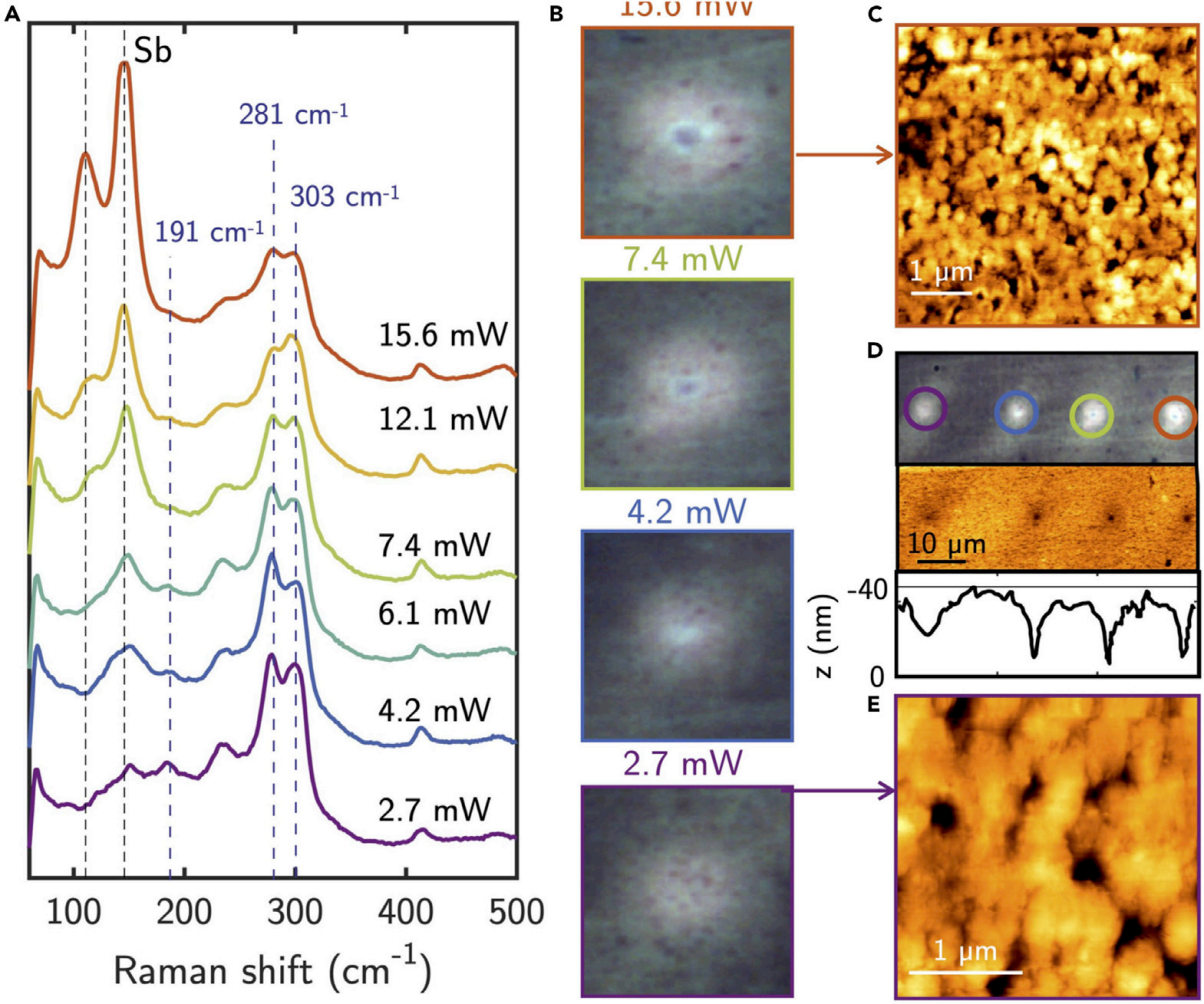
Structural and morphological analysis of laser crystallized Sb_2_S_3_ (A) Raman spectra of laser crystallized Sb_2_S_3_ using a 532-m laser at different power ranging from 2.7 to 15.6 mW. (B) Micrographs of the laser crystallized spots, showing ablation of Sb_2_S_3_ at the center of the laser Gaussian profile. AFM topography of laser crystallized regions at power of (C) 15.6 and (E) 2.7 mW. (D) AFM topography of laser crystallized spots with line profiles showing the ablation of Sb_2_S_3_ at the center of the laser spot.

Laser crystallization has also been investigated using green CW lasers (514 and 532 nm) with wavelengths above the absorption edge of both c-Sb_2_S_3_ and a-Sb_2_S_3_, operated at different power. Raman spectra taken after laser irradiation at increasing power are shown in Figure 8. Comparing the Raman spectrum for the lower power of 2.7 mW with those of *Type-I* and *Type-II* thermally crystallized samples, it can be noticed that, statistically, the A_g_(303cm^−1^)/A_g_(281cm^−1^) < 1 and the modes below 150 cm^−1^ are predominant than the 191 cm^−1^ mode, similarly to the Raman spectra of *Type-II* bright region, as also confirmed by the AFM morphology shown in Figures 8C–8E, which is similar to the morphology reported in the inset of Figure 4C. Therefore, we can infer that laser crystallization results in *Type-II*-like bright regions, consistent with the fact that the segregation of Sb clearly appears by increasing the laser power, similarly to the increase of temperature above 300–350°C. Optical and AFM images in Figures 8B–8D also show that for laser powers higher than 7.4 mW, ablation of Sb_2_S_3_ at the center of the laser’s Gaussian intensity profile occurs, indicating that the local temperature may exceed 450°C. At *T* > 450°C, we have also observed thermally the loss of samples’mass, with the complete loss of the Sb_2_S_3_ at *T* ∼ 550°C (Makreski et al., 2013). This underlines the difficulty to reach the melting temperature of Sb_2_S_3_ of 540°C without the loss of material, which could be a challenge for the reproducibility over a large number of cycles of Sb_2_S_3_ phase change.

## Discussion

**Figure 9:**
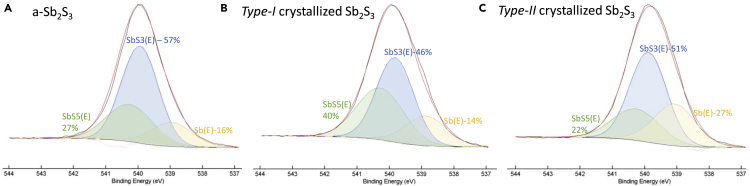
Chemical XPS analysis of crystallized Sb_2_S_3_ High-resolution XPS spectra of the Sb_3d3/2_ photoelectron core level of (A) a-Sb_2_S_3_, (B) *Type-I*, and (C) *Type-II* crystallized Sb_2_S_3_. The spectra were fitted with three components corresponding to the SbS_3_(E) and SbS_5_(E) units and Sb(E) electrons lone-pair.

Sb_2_S_3_ crystallization by thermal annealing and green laser irradiation has highlighted two main regimes and structures. A deeper insight into those regimes can be further gained by the XPS analysis reported in Figure 9. Specifically, the fitting of the Sb_3d3/2_ photoelectron core level indicates three main states of Sb, namely the Sb^+5^ at binding energy (BE) of 540.3 eV owing to the SbS_5_(E) units, the Sb^+3^ at BE = 539.9 eV owing to the SbS_3_(E) units, and the Sb-electrons lone-pair at the low BE = 538.9 eV (Stamenković et al., 2021). The a-Sb_2_S_3_ has a certain relative volume fraction of those units that of course depends on the disorder induced by the deposition method and conditions, with a predominance of SbS_3_(E) units in our case as inferred by the higher Sb^+3^ fitting component (the perfect c-Sb_2_S_3_ has 50% of SbS_3_(E) and SbS_5_(E) units – see Figure 1). Interestingly, *Type-I* crystallized samples show a ratio of the SbS_5_(E)-(40%) and SbS_3_(E)-(46%) units approaching the ideal 1:1 ratio of c-Sb_2_S_3_, consistent with the Raman A_g_(303cm^−1^)/A_g_(281cm^−1^) ∼ 1 (see Figure 4). Conversely, Figure 9 indicates that *Type-II* crystallized samples show a ratio of the SbS_5_(E)-(22%) and SbS_3_(E)-(51%) units lower than 1, consistent with the Raman A_g_(303cm^−1^)/A_g_(281cm^−1^) < 1 (see Figure 5). Therefore, we can infer that thermal annealing at 250°C < *T* < 300°C yields a crystallized structure with SbS_5_(E) and SbS_3_(E) units close to the single crystal structure with large spherulitic crystals as in Figures 4C and 4D, resulting in the absence of the oxidation or degradation of this kind of crystallized structure during the phase transformation. This also supports the use of *Type-I* Sb_2_S_3_ without any capping layer, as in the present study.

Conversely, *Type-II* crystallization by thermal annealing at *T* > 300°C or laser irradiation, because of some loss of sulfur, results in a reduced presence of SbS_5_(E) units and the appearance of Sb-segregation that further oxidizes.

However, the possibility to switch between contrasts, i.e., *Type-I* and *Type-II* in controlled manner gives extra freedom in terms of photonic memory and the devices.

Those two identified regimes, and the negligible oxidation at *T* < 300°C, are consistent with a previous study on the kinetics of the oxidation of Sb_2_S_3_ at temperatures from 300°C up to 500°C, i.e., below its melting temperature (Padilla et al., 2014). Specifically, oxidation of Sb_2_S_3_ starts at 350°C and its rate increases by more than two orders of magnitude to 500°C, staying negligible in an N_2_/O_2_ atmosphere at *T* < 250°C. As supported by the change of the S/Sb ratio by XPS ad EDX, the oxidation at *T* > 350°C is due mainly to the loss of sulfur, and mainly occurs at the surface of Sb_2_S_3_, whereas *Type-II* crystallization homogenously involves the rest of the film depth. In this direction, crystallization could be improved even at *T* > 350°C by a capping layer, although this study is still ongoing and requires optimization. Conversely, for GST, the oxidation is more problematic and related to the different reactivities of Ge, Sb, and Te, i.e., the oxygen exposure leads to a depletion of Te from GST surface and heterogeneous formation of the Sb- and Ge-oxides, with Sb-oxides more in depth and Ge-oxides more toward the surface (Golovchak et al., 2015).

### Reconfigurable nanophotonic phase modulator on-chip

High-performance, low-loss, compact reconfigurable photonic devices on-chip are essential building blocks of modern adaptive optical network (Cheng et al., 2018; Feldmann et al., 2019, 2021). Compact and energy efficient phase modulator represent the first evolutionary step from reconfigurable devices into programmable circuits.

**Figure 10:**
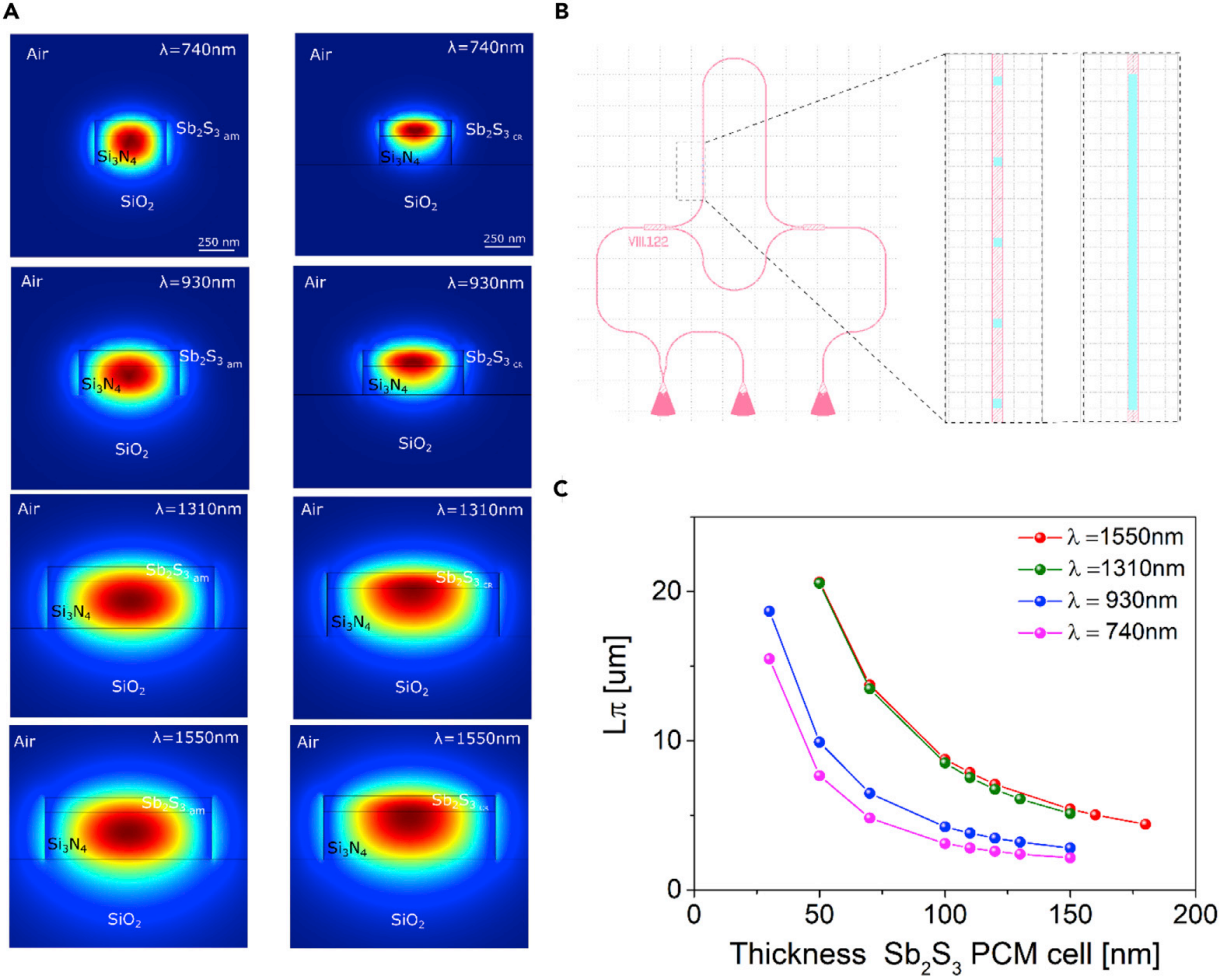
Design ad simulation of MZI phase modulator with Sb_2_S_3_ PCM cell (A) Simulated guided mode profile through the Si_3_N_4_ waveguide with a-Sb_2_S_3am_ and c-Sb_2_S_3_ (*Type-I*) PCM cell h = 110nm atop. Si_3_N_4_ waveguide dimensions are determined to support single TE_0_-like mode, namely at λ = 740 nm: *h* = 200 nm, *w* = 500 nm; λ = 930 nm: *h* = 200 nm, *w* = 700 nm; λ = 1,310 and 1,550 nm: *h* = 334 nm, w = 1,200 nm; at λ = 1,550: neff_am_ = 1.64106, neff_cr_ = 1.739502. (B) The design of MZI phase modulator equipped with PCM cell atop one of the arms in two configurations: patterned cells and unpatterned cell. (C) PCM length L_π_ needed to attain π-shift of the mode in the arm equipped with the cell atop as a function of thickness of PCM cell for different wavelength of the guided mode.

Thus, our goal is the realization of low-loss nanophotonic phase modulator on-chip based on pioneer-investigated PCM cell transition a-Sb_2_S_3_ ↔ c-Sb_2_S_3_. Controllable optical modulator is based on Si_3_N_4_ MZI on-chip, where the input optical signal splits into two arms by multimode interference (MMI) junction, after a certain distance both modes recombine at the output MMI combiner in a single arm producing the interference of both beams as in Figure 10B. Output intensity of the signal depends on the phase difference between optical modes in two branches. The establishment of reconfigurable PCM cell atop one of the arms of MZI results in the relative phase shift of the carried optical signal.

The design of the established nanophotonic device is based on the numerical investigation of the evanescent interaction between the transverse electric (TE_0_) mode in the waveguide and integrated PCM cell atop it. The reversible switching of PCM phase state generally can be triggered by appropriate heat stimulation via evanescent part of intense guided mode (Feldmann et al., 2019, 2021; Ríos et al., 2015; Stegmaier et al., 2017) or by help of extra low-loss microheaters deposited in the vicinity of PCM cell (Wu et al., 2019; Zheng et al., 2020b, 2020a). Joule heating elevates the temperature of PCM cell above the melting point following quenching results in an amorphous phase-state, whereas an increase in the temperature above the glass transition results in a crystalline phase-state.

The modeled evanescent interaction between the nanoguide mode and atop Sb_2_S_3_ cell in the amorphous and crystalline states are shown in Figure 10A. We benefit from utilization Sb_2_S_3_ in the visible and infrared (IR) region owing to negligible optical loss of both states and good optical contrast (Figure 7B). Therefore, we consider reconfigurable MZI phase modulators equipped with PCM cell for wide application range modulation of the signal in the visible and IR region, in particular at telecommunication band wavelength λ = 1,310 nm where standard optical fiber exhibits zero chromatic dispersion and at telecommunication wavelength λ = 1,550 nm obtaining minimum optical propagation loss. Upon phase transitions a-Sb_2_S_3_ ↔ c-Sb_2_S_3_, the guided mode is lifted up owing to the refractive index change of PCM resulting in the modification of the mode phase.

The essential parameter to be determined is required PCM length L_π_ needed to attain π-phase difference between optical modes in both branches as a result of the switching between cell states thus ensuring amplitude key-switch (switching on/off) of the output signal with corresponding extinction ratio at necessary wavelength. Simulation results are shown in Figure 10C, where owing to higher contrast of the refractive index of amorphous and crystalline states of Sb_2_S_3_ at visible wavelength range required thickness of PCM cell is lower to obtain π-shift in comparison with IR guided modes.

**Figure 11:**
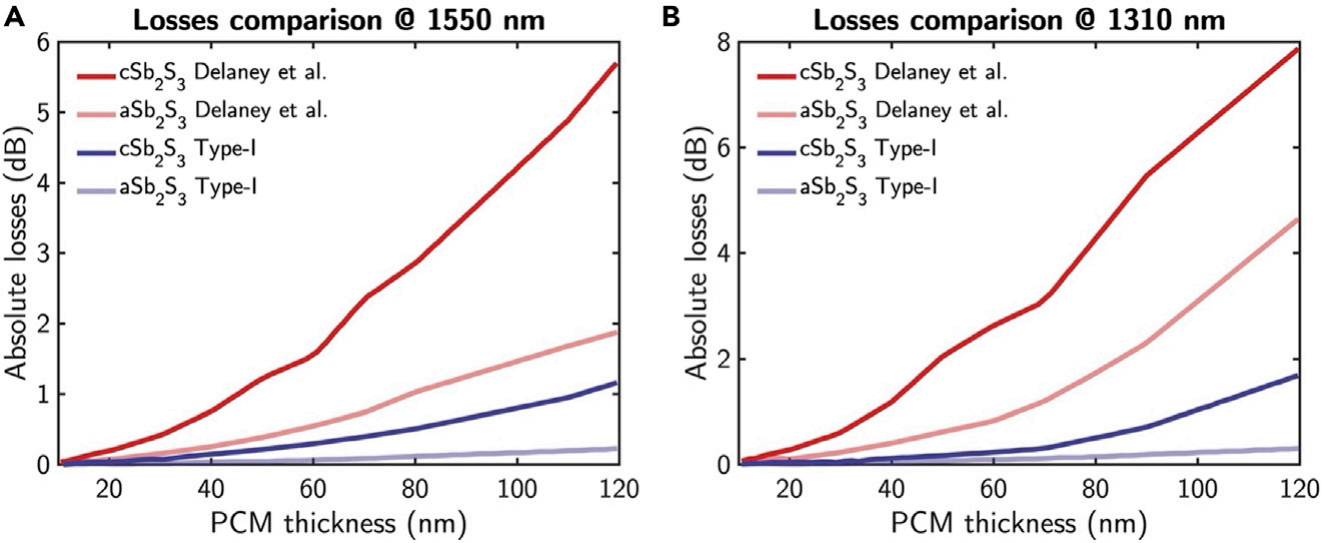
Analysis of losses for a Sb_2_S_3_-based MZI phase modulator Comparison between Type-I (blue) and Delaney et al. (2020) (red) models of the Interface losses at (A) 1,550 and (B) 1,310 nm varying the deposition thickness of the amorphous (dashed line) and crystalline (dotted line) Sb_2_S_3_ over the designed waveguide in Figure 10.

Figure 11 shows the numerically simulated interface losses varying the thickness of the deposited Sb_2_S_3_ at 1,550 and 1,310 nm. In the figure, the results by the optical properties for the amorphous and crystalline *(Type-I)* Sb_2_S_3_ from this study (Blue lines) and Delaney et al. (2020) (Red lines) are compared. The numerical results indicate lower interface losses for the present work optical constants by a factor of opening the possibility of integrating the phase modulator, based on ${\text{Sb}}_{2}{\text{S}}_{3}$, in a programmable SiN photonic platform.

Further improvement, namely a decrease of two times L_π_, can be reached by placing PCM cells in both arms of MZI and antipolar switching of both cells (PCM cell in the one arm in the amorphous state, whereas PCM cell in another arm – in the crystalline state and vice versa), which can be obtained by utilization controllable individual microheaters (Ovvyan et al., 2016).

Another improvement strategy of the switching the states of PCM cell is patterning it into subwavelength-nanostructures atop the center of the waveguide above the maximum electric field of the nanoguide mode (Figure 10B) following in highly uniform heating of each micro-cell, resulting in an increase of the switching contrast and a decrease of required PCM area in comparison with the unpatterned cell (Wu et al., 2019).

Introducing PCM cell atop ring resonator on-chip establishes phase modulator and optical switch (Fang et al., 2021; Stegmaier et al., 2017).

Further potential application of explored PCM material is the introduction of a phase-gradient Sb_2_S_3_ metasurface atop photonic device, resulting in programmable broadband mode converter for realization unique scalable optical functionalities (Karvounis et al., 2016; Li et al., 2017; Wu et al., 2021).

The cycling endurance of Sb_2_S_3_ is a matter of ongoing investigation and optimization worldwide. Presently, from literature, the cycling record reported for Sb_2_S_3_ varies from 30 cycles for a phase change degree (PCD) of 90%–7,000 cycles for PCD of 20% (Gao et al., 2021), clearly indicating that work is needed to control the crystallization of Sb_2_S_3_ and consequently the cycles. In the case of GST, no considerable degradation is detected experimentally after 1,000 cycles (Stegmaier et al., 2017) without extra optimization of the cell-design and its operation scheme. The number of operation cycles is eventually limited by the endurance of the PCM cells, thus individual GST devices in endurance experiments have shown 10^12^ switching cycles (Kuzum et al., 2012). Noteworthy, the number of cycles is not the only relevant parameter to consider.

Indeed, it should be noted that the absorption losses of both amorphous and crystalline phases of GST PCM cell leads to fundamental limitation phase modulation circuits, namely it prevents optical phase control independently of changes in the amplitude of propagated light through the photonic devices in the telecommunication band. Therefore, one of the ways to build efficient lossless phase modulator based on integrated PCM cell on one of the arms of the MZI interferometer is Sb_2_S_3_, which shows extremely low insertion loss (*k*, is less than 10^−5^), whereas remaining non-volatile at operating temperatures, where crystallization and amorphization temperature are comparable with GST.

Furthermore, another potential benefit of Sb_2_S_3_ is the existence of two crystalline states of Sb_2_S_3_ (*Type-I* and *Type-II*) explored in this work, which enables the multilevel operation of the phase modulator as well as provides extra degree of freedom of non-volatile memory enabling applications of hybrid photonic devices with integrated PCM cells.

### Design and modeling of an Sb_2_S_3_ as high refractive index dielectric reconfigurable antenna

**Figure 12:**
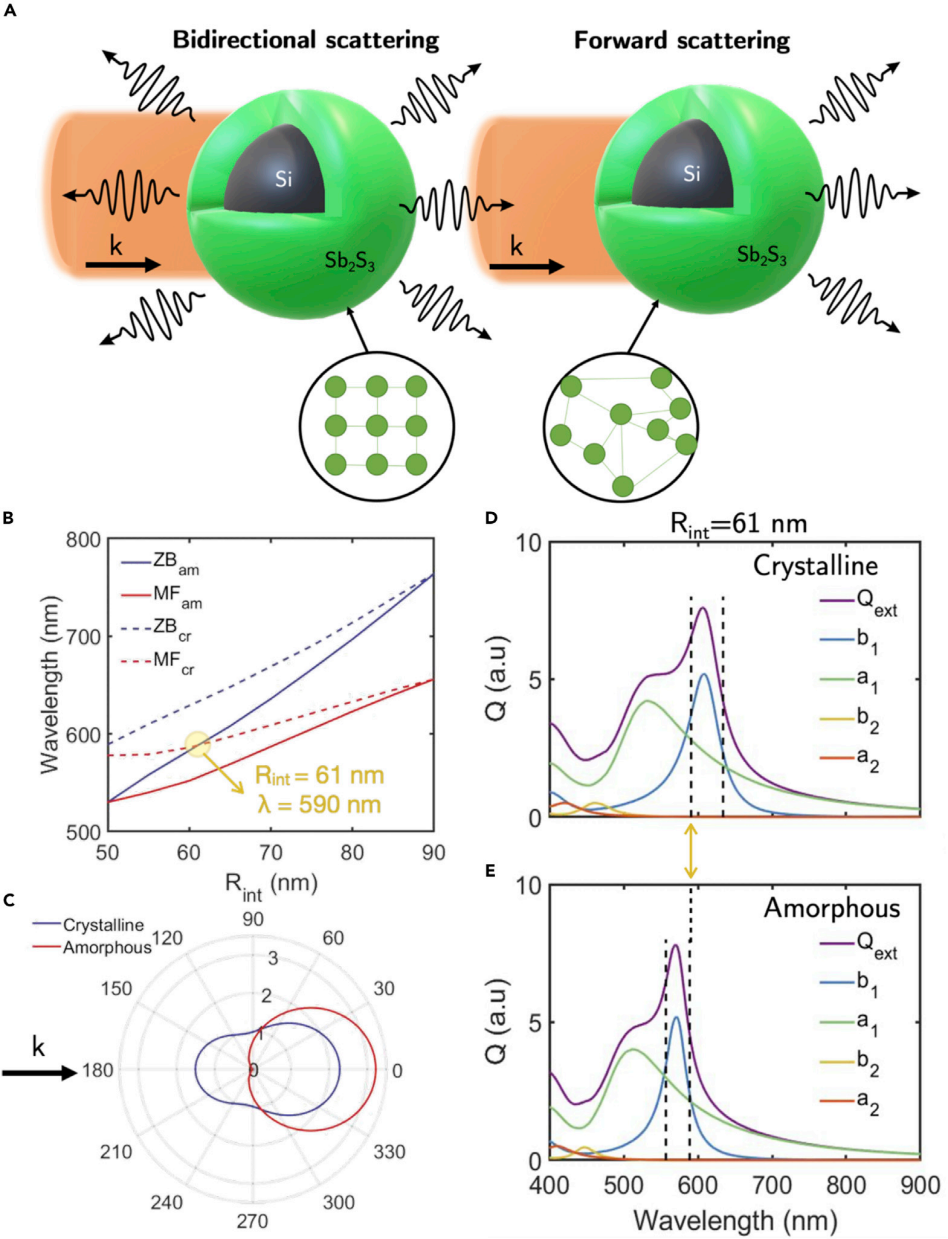
Design, simulation, and Scattering diagrams for a reconfigurable Si-core/Sb_2_S_3_-shell nanoantenna (A) Sketch of the proposed Si-core/Sb_2_S_3_-shell nanoantenna working for the amorphous and crystalline (*Type-I*) phases of Sb_2_S_3_. (B) Evolution of the Kerker conditions (ZB and MF) for both phases of Sb_2_S_3_ as a function of the exciting wavelength and of the nanoantenna internal radius. The external radius is fixed to 90 nm. For an internal radius of 61 nm and an incident wavelength of 590 nm, MF condition for the crystalline phase and ZB condition for the amorphous phase coincide (see the text for details). (C) Scattering diagrams for an incident wavelength of 590 nm, *R*_1_ = 61 nm, *R*_2_ = 90 nm, for both phases (crystalline and amorphous). For the crystalline phase, light is scattered in both directions, whereas for the amorphous phase, most of the radiation is scattered in the forward semi space direction (black arrow indicates the direction of the incident wavevector). Extinction efficiency of the core-shell nanoparticle for the Sb_2_S_3_ (D) amorphous phase and (E) crystalline phase. In (D) and (E), the dashed lines indicate the coherence points between the electric and magnetic dipole contributions.

Dielectric nanoparticles, with a high refractive index (HRI), are able to locate and intensify electromagnetic fields with Joule losses almost null. For characteristic incident wavelengths, confined displacement currents lead to resonances known as whispering gallery modes. These can be understood as rays enclosed and traveling in a spherical cavity supported by multiple total internal reflections. According to the Mie theory, these resonances can be either of electric or of magnetic character and can be independently excited by changing the incident wavelength. For some situations, they can overlap in certain spectral regions, leading to coherent effects between the scattered electromagnetic fields. These effects produce characteristic angular distributions of the scattered electromagnetic energy; hence, HRI nanoparticles can be used to control the direction of the scattered radiation. When those coherent effects are predominantly between electric and magnetic dipole resonances, interesting directionality phenomena can be found, like a minimum scattering in the forward direction or a null backscattered intensity phenomenon. In the literature, these are known as the Kerker conditions (Kerker et al., 1983), light is scattered predominantly either in the forward direction, being null in the backward direction (first Kerker condition or zero backward (ZB)) or mostly in the backscattered direction, being minimum in forward (second Kerker condition or minimum forward (MF)). In general, there are other coherent effects owing to interference with higher order resonances that lead to a wider gamut of directionality effects (Tribelsky et al., 2015). Consequently, HRI dielectric nanoparticles can be considered as nanoantennas whose radiation directionality can be controlled depending on the incident wavelength, the external medium, and the geometry of the nanoparticle (Geffrin et al., 2012). Here, a core-shell geometry schematized in Figure 12 is analyzed, with the core made of silicon (a conventional HRI material in the NIR; García-Etxarri et al., 2011) and the shell made of Sb_2_S_3_ as PCM, whose optical properties depend on its phase (amorphous or crystalline) as described in Figure 7. Depending on the phase of Sb_2_S_3_, the direction of the scattered light can be either in the forward or in a mixture of both directions (see Figure 12A). A core-shell geometry is selected to have enough degrees of freedom to obtain the suitable conditions to generate the desired directionality effects, as the optical properties of the phases of Sb_2_S_3_ do not permit by themselves to get these conditions from a single nanoantenna made only of this material.

The internal and external radii have been optimized to get directional switching of the scattered radiation. Here we consider the external radius of 90 nm and the internal radius of 61 nm. The first and second Kerker conditions for both phases (either amorphous or crystalline) are plotted as a function of the nanoantenna internal radius and the incident wavelength in Figure 12B. For an internal radius of 61 nm and an incident wavelength of 590 nm, both MF and ZB conditions coincide for both phases. Therefore, by fixing these parameters, a reconfigurable nanoantenna could be designed with the scattering diagram for an incident wavelength of 590 nm shown in Figure 12C. For the crystalline phase, radiation is scattered in both forward and backward directions, whereas for the amorphous phase, light is scattered predominantly in the forward semi space direction, being almost null in the backscattered direction. It is important to point out that Kerker conditions are only valid when coherent effects between the electrical and magnetically dipole resonances are predominant. In this case, higher order resonances have to be considered (in this case, the quadrupolar contributions is not negligible. See Mie coefficients a_2_ and b_2_ (Bohren and Huffman, 1998) in Figure 12D. Nevertheless, a very efficient switching effect in the backward direction can be achieved for these optimized parameters by controlling the crystallinity of Sb_2_S_3_. Figures 12D and 12E show the extinction efficiency (*Q*_ext_) together with the electric and magnetic dipole modes contribution (a_i_ and b_i_ Mie coefficients; Bohren and Huffman, 1998) for the amorphous and crystalline phases, respectively.

**Figure 13:**
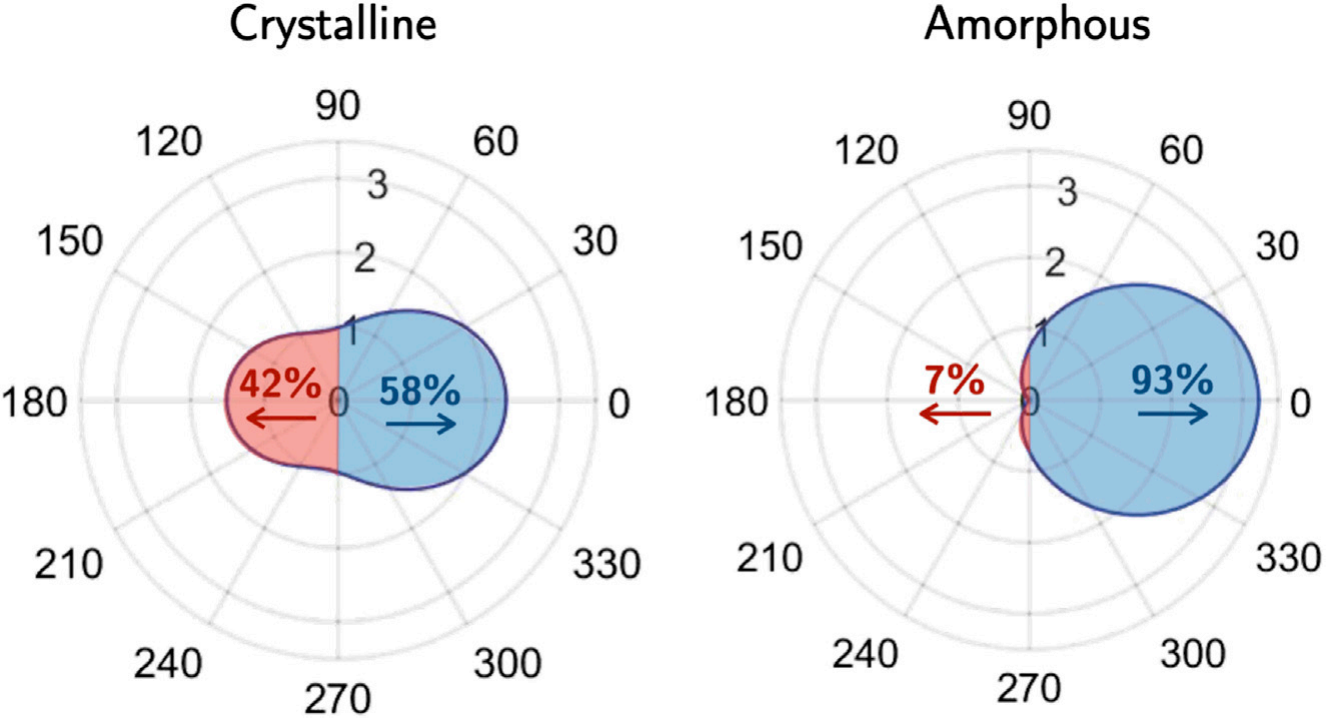
Scattering angular diagrams of Si-core/Sb_2_S_3_-shell nanoantennas Scattering angular diagrams of the proposed core-shell nanoparticle for an incident wavelength of 590 nm, for an internal and external radius of 61 and 90 nm, respectively, and for the crystalline and amorphous phases of Sb_2_S_3_. The percentage of scattered light in both forward and backward directions, for both phases, is shown.

The angular scattering diagrams of the proposed core-shell nanoantenna are plotted for the two phases of Sb_2_S_3_ in Figure 13. In this case, the intensity of the scattered radiation has been integrated for every scattering angle to calculate the percentage of radiation scattered in either the forward semi space or the backward semi space, as indicated in Figure 13 by arrows. A switching effect by changing the Sb_2_S_3_ phase is clearly obtained in the forward and backward directions. For the former, we get a contrast ratio Am/Cr of 2 (3 dB), and for the latter, we get a contrast ratio of 0.17 (8 dB). Furthermore, it is important to point out that for the crystalline phase, the nanoantenna acts as a “beam splitter” by dividing the scattered power between the forward and backward semi-spaces.

### Limitations of the study

The amorphous/crystalline cycle endurance of Sb_2_S_3_ has not been measured here. This study does not make claims about the reversibility over a high number of cycles of phase changes. Based on our methodology, we analyze statistically the structural and corresponding optical changes only over one cycle, as after the reported crystallization, we went back to the same Rama spectra of the amorphous phase.

## STAR★Methods

### Key resources table

**Table:** 

REAGENT or RESOURCE	SOURCE	IDENTIFIER
c-Sb2S3	2D Semiconductor	https://www.2dsemiconductors.com/sb2s3/
c-Si (100)	MTI	https://www.mtixtl.com/Si-P-a-101D05C2-US.aspx
Sapphire substrate	PIKEM	G200201
Glass slide	CORNING	7059

### Resource availability

#### Lead contact

Further information and requests for resources and reagents should be directed to and will be fulfilled by the Lead Contact, Maria Losurdo (maria.losurdo@cnr.it).

#### Materials availability

All materials used and generated in this study will be made available on request from the Lead Contact with a completed Materials Transfer Agreement.

### Experimetal model and subject details

#### Sb_2_S_3_ materials details

Sb_2_S_3_ single crystals, c-Sb_2_S_3_, were purchased from 2D semiconductors.

Amorphous Sb_2_S_3_ ,a-Sb_2_S_3_, films were deposited by chemical bath deposition (CBD) (Cobianu et al., 2021) using 6.5 g of AR grade, anhydrous antimony chloride (SbCl_3_) dissolved in 33.5 mL of methanol and dissolving 46.5 g of sodium thiosulfate (Na_2_S_2_O_3_ x 5H_2_O) in 150 mL of deionized (DI) water (1.25 M solution). These two solutions were mixed at a constant temperature of 15°C, and then DI water was added to obtain a total volume of 750 ml of solution with a pH = 4.

a-Sb_2_S_3_ films were deposited on 4″ Si(100) wafers, 2″ sapphire wafers and glass slides, in order to check any effect of the substrate on the a-Sb_2_S_3_ structure. The substrates were cleaned in 5% sodium hydroxide at 90°C followed by 1N HCl, and absolute ethanol. After rinsing in DI water, the substrates were dried at 80°C and then immersed in a vertical position in a beaker. The growth time was between 1h - 4h to have a film thickness in the range 100 nm to 1μm.

For the interlaboratory study, two batches of samples of a-Sb_2_S_3_ with a thickness of approximately 150 nm on various substrates of glass, sapphire, Si(100), were analyzed at the various laboratories of the co-authoring institutions. The same samples were thermally crystallized at the various laboratories in atmosphere of argon, air and under vacuum; for the thermal annealing temperatures in the range 250 - 350°C were investigated. Temperatures higher than 350°C resulted in strong changes of stoichiometry and loss of mass of films at T>450°C.

Additionally, the same set of a-Sb_2_S_3_ samples, without any capping layer, was also exposed to green laser irradiation (514 nm and 532 nm) to assess the laser crystallization of the films. A laser power in the range 2.7 – 15.6 mW was investigated with a laser spot diameter of ≈1 μm.

### Method details

#### Chemical and morphological characterizations

Scanning electron microscopy (SEM) was carried out for the morphological characterization of the samples with a Zeiss Supra 219 40 FEG SEM equipped with a Gemini field emission gun. Energy-dispersive-X-ray analysis (EDX) was used to check elemental composition of the films. Analyses were carried out at an extraction voltage of 3 kV and 221 a 30-μm aperture.

The elemental composition was cross-checked by x-ray photoelectron spectroscopy (XPS). XPS measurements were carried out by a Scanning XPS Microprobe (PHI 5000 Versa Probe II, Physical Electronics) equipped with a monochromatic Al Kα x-ray source (1,486.6 eV), with a spot size of 200 μm. Survey (0–1,200 eV) and high-resolution spectra (C1s, S2p, S2s, Sb3d) were recorded in FAT mode at a pass energy of 117.40 and 29.35 eV, respectively. Spectra were acquired at a take-off angle of 45° with respect to the sample surface. Surface charging was compensated using a dual beam charge neutralization system, and the hydrocarbon component of C1s spectrum was used as internal standard for charging correction, and it was fixed at 285 eV.

Morphology was investigated by atomic force microcopy (AFM) in the interlaboratory study. The topography images were acquired in three different laboratories using two instruments of AFM NX10 from Park Systems in non-contact mode using PPP-NCHR tip probe with radius of curvature less 10 nm, and Autoprobe CP (Thermomicroscope) used also in non-contact single-pass mode using a gold-coated Si tips (their frequency is ∼80 Hz).

#### Raman spectroscopy

In the interlaboratory study, five different Raman setups in confocal configuration were used at different laboratories for unpolarized Raman spectra acquisition. (i) A LabRam Horiba instrument operating with a 532 nm wavelength laser and a ×100 microscope objective (NA = 0.9). (ii) A Invia Reflex Renishaw Raman instrument was used operating with a 532 nm wavelength laser and a ×100 microscope objective (NA = 0.9). (iii) A Invia Reflex Renishaw Raman instrument was used operating with a 514 nm wavelength laser and a ×100 microscope objective (NA = 0.9); (iv) A Xploraplus Horiba Raman operating with 532 nm laser and a ×100 microscope objective (NA = 0.9); (v) A Horiba LabRam Aramis VIS operating at 532 nm with a×100 microscope objective (NA = 0.9) was used. The size of the laser spot on the sample was approximately 1 μm.

For the statistical analysis of the interlaboratory measurements ten random Raman spectra for each sample were taken in each laboratory. The laser power was 0.2 – 2 mW for the characterization f materials without inducing any phase change.

#### Spectroscopic ellipsometry

In the interlaboratory study, two different spectroscopic ellipsometers were used for the optical characterization at the various laboratories. (i) A phase-modulated spectroscopic ellipsometer (UVISEL Horiba) operating in the 0.75 – 6.0 eV energy range with a resolution of 0.05 eV and with the angle of incidence varying in the range 55°-70°. (ii) A J.A. Woollam VASE M-2000 spectrocopic ellipsometer operating in the spectral range 193 nm to 1690 nm at variable angles from 55 to 75°. All measurements were performed at ambient conditions and at room temperature.

Ellipsometric spectra of the pseudodielectric function were analyzed by a simple isotropic model substrate/Sb_2_S_3_/surface roughness. The substrate was experimentally measured before the deposition; the surface roughness was modelled by a Bruggeman effective medium approximation (BEMA) of 50% Sb_2_S_3_+50%voids with the thickness set at the RMS value from AFM.

The a-Sb_2_S_3_ was parameterized with a Tauc-Lorentz dispersion equation (Jellison and Modine, 1996); the Tauc-Lorentz parameters and the thickness of the film were the fit parameters.

The crystallized Sb_2_S_3_ films were parameterized with an ensemble of Tauc–Lorentz oscillators for the different electronic transitions of Sb_2_S_3_ as in Figure 2. The best fit was obtained by three oscillators, representing main CPs associated to interband transitions as described in main text. The values of E_gap_ and E_n_ obtained from the best fit of the ellipsometric spectra for the films are summarized in the Table below.

**Table:** Tauc-Lorentz parametrization of Type I and II thermalized crystallize Sb_2_S_3_

	*a-* Sb_2_S_3_	*Type I*	*Type II*
E_gap_ (eV)	2.20 ± 0.05	1.6 ± 0.1	1.4 ± 0.1
ε_∞_	1.5 ± 0.1	2.4 ± 0.1	2.1 ± 0.1
A_1_	133.8 ± 0.4	37.1 ± 0.6	23.0 ± 0.5
E_1_	3.78 ± 0.05	2.20 ± 0.05	2.41 ± 0.05
C_1_	5.40 ± 0.09	2.2 ± 0.4	1.0 ± 0.1
A_2_		17.1 ± 0.6	15.2 ± 0.4
E_2_		3.61 ± 0.03	3.1 ± 0.05
C_2_		1.9 ± 0.3	1.5 ± 0.4
A_3_		24.6 ± 0.4	4.1 ± 0.6
E_3_		5.51 ± 0.06	5.82 ± 0.07
C_3_		2.8 ± 0.2	2.4 ± 0.3

#### Polarimetry

Polarimetric measurements were performed with an adapted microscope set-up (Nikon Eclipse LV-N). The microscope base structure includes a monochromatic unpolarized light source (CoolLED pE-100, 633 nm), a polarizer (P1) before de sample holder, objectives (5x, 20x and 50x with NA of 0.15, 0.4 and 0.6 respectively) and an analyzer (P2) before the CCD camera (The Imaging Source DMK 33UX174). To achieve the measurement of the Mueller Matrix (MM) with the microscope two λ/4 waveplate retarders (R1 and R2) with controllable angular orientation of the fast axes were included. One after P1 and another before P2. Both retarders are identical (Thorlabs WPH05M-633) and are mounted onto motorized rotation stations (Thorlabs PRM1/MZ8) controlled by an ordinary laptop. We worked out a MATLAB routine that controls the angular position of R1 and R2 fast axis synchronously with the acquisition process of the camera. The routine consisted in measuring N images, with angular increments in the fast axis orientation of 180°/N for R1 and 900°/N for R2 (5:1 speed ratio) between each image acquisition. As a result, in each pixel of the camera we measured a full Fourier measurement cycle that was processed to obtain the corresponding MM of a given part of the sample. As a result, a polarimetric image is obtained, i.e., an image that describes with spatial resolution what changes are produced in the light polarization by a sample. The full process (data acquisition plus post processing) took less than 20 minutes.

A differential analysis of the Mueller matrices using the Mueller Matrix Differential Decomposition (MMDD) was performed (Arteaga and Kahr, 2013). The differential matrix m relates the Mueller matrix M as

(Equation 2) $$\frac{dM}{dz}=\mathrm{mM}$$

If the differential matrix, m does not depend on z, i.e., distance travelled along the direction of propagation, the solution of this differential equation is found by taking the logarithm of M, thus,

(Equation 3) m=ln(M)

The most general form of the differential Mueller matrix for a non-depolarizing medium is given as follows (Azzam, 1978; Ossikovski, 2011; Arteaga and Kahr, 2013)

(Equation 4) $$m=\left(\begin{array}{llll} \alpha & -LD & -LD^{\prime } & CD\\ -LD & \alpha & CB & LB^{\prime }\\ -LD^{\prime } & -CB & \alpha & -LB\\ CD & -LB^{\prime } & LB & \alpha \end{array}\right)$$

where LD, LD′ and CD are linear dichroism in the *x-y* axes, linear dichroism in the 45-135° and circular dichroism. LB, LB′ and CB stand for linear birefringence in the *x-y* and 45-135° axes and circular birefringence, respectively. The value α is the absorption coefficient.

#### First principles calculations

Density functional theory first principles calculations based on a numerical atomic orbital method were carried out using SIESTA code(Soler et al., 2002). All the calculations were performed with the van der Waals density functional scheme proposed by Dion et al. (2004) implemented in SIESTA (Román-Pérez and Soler, 2009) to simulate the electronic exchange and correlation. Core electrons are described by ab initio optimized norm conserving pseudopotentials, generated following the recipe given by Hamann (2013), available in the PSEUDODOJO(van Setten et al., 2018) in the Kleinman–Bylander fully nonlocal separable representation. The 4*d*, 5*s* and 5*p* electrons were considered as valence electrons of Sb and explicitly included in the calculations. For S, as valence electrons, 3*s*, 3*p*, and 3*d* were chosen.

The one-electron Kohn Sham eigenvectors were expanded in a basis of localized numeric atomic orbitals (NAO) as implemented in SIESTA code. The size of the basis set chosen for Sb was simple ζ for the semicore 4*d*; double ζ for the 5*d* polarization orbital and triple ζ for the 5*s* and 5*p* shells. The size of the basis set chosen for S was double ζ for 3*d* polarization orbital; triple ζ for the 3*s* and 3*p* shells. In all cases the default cutoff radii of the strictly localized wave function were employed, including a soft confinement potential to reproduce the decaying tails of the functions. The electronic density, Hartree, and exchange correlation potentials, as well as the corresponding matrix elements between the basis orbitals, were calculated in a uniform real space grid. The equivalent plane wave cut-off used to represent the charge density was 1000 Ry. For the Brillouin integrations, a Monkhorst–Pack (Monkhorst and Pack, 1976) sampling of 6 × 18 × 6 was used. For the bulk structure computation, atoms and unit cell were allowed to relax until the maximum component of the force acting on any atom was smaller than 0.01 eV Å^−1^, and the maximum component of the stress was 0.1 GPa.

##### Optical response

The frequency dependent optical response of the studied structures was obtained using first-order time-dependent perturbation theory to calculate the dipolar transition matrix elements between occupied and unoccupied single-electron eigenstates as implemented in SIESTA code. The optical constants of a solid was derived from the complex dielectric function $\varepsilon \left(\text{ω}\right)=\varepsilon_{2}\left(\text{ω}\right)+\text{i}\varepsilon_{2}\left(\text{ω}\right)$. The frequency-dependent dielectric function can be written within the dipole approximation as

$$\varepsilon_{2}\left(\text{ω}\right)=\frac{2\text{π}}{\text{mN}}\frac{{\text{ω}}_{\text{p}}^{2}}{{\text{ω}}^{2}}\sum_{\text{v},\text{c}}\int_{\text{BZ}}\frac{\text{dk}}{{\left(2\text{π}\right)}^{3}}{\left|{\text{M}}_{\text{cvk}}\right|}^{2}\text{δ}\left({\text{ε}}_{\text{ck}}-{\text{ε}}_{\text{vk}}-\hbar \text{ω}\right)$$

where m is the electron mass, N is the number of electrons per unit volume, and ${\text{ω}}_{\text{p}}^{2}=4{\text{Ne}}^{2}/\text{m}$ is the plasma frequency, with e being the electron charge. The single particle electronic states |ψ of energy ε were labeled by their crystal momentum k and their valence (V) and conduction (c) band index. The sum was over connecting valence and conduction states and over all the k points in the first Brillouin zone. The optical matrix element was given by ${\text{M}}_{\text{cvk}}={\text{ψ}}_{\text{ck}}\left|\hat{\text{e}}\cdot \text{p}\right|{\text{ψ}}_{\text{vk}}$, where $\hat{\text{e}}$ is the polarization of the incident light and p is the momentum operator. The real part of the dielectric function $\varepsilon_{1}\left(\text{ω}\right)$ was obtained from the imaginary part using the Kramers-Kronig relation.

In order analyze the origin of the peaks appearing in the spectra due to interband transitions, the values of the optical matrix element ${\text{M}}_{\text{cvk}}$ for every pair of conduction and valence bands at each k point with an energy difference equal to the photon energy at which the peak appear were calculated. In this way, the pair of bands contributing to the interband transition visible in the $\varepsilon_{2}\left(\text{ω}\right)$ spectra were analyzed. Convergence test were carried out to determine the number of bands included in optical calculations. The optical mesh used was 4 × 12 × 4. The gaussian broadening was set to 0.2 Ry. Optical response parallel to each of the crystallographic axes was calculated using the polarized type, which considers the application of the electric field in the given direction.

### Quantification and statistical analysis

Ellipsometric fitting was performed using DeltaPsi2 software. Electromagnetic simulation of the on chip modulator and nanoantenna were performed using Ansys Lumerical FDTD. Figures were produced with MATLAB R2020a from the raw data.

### Additional resources

Any additional information about the film fabrication, annealing, tests and data reported in this paper is available from the lead contact on request.

## Data Availability

• Data reported in this paper will be shared by the lead contact upon request. • This paper does not report original codes. • Any additional information required to reanalyze the data reported in this paper is available from the lead contact upon request.
