# Interlaboratory study on Sb_2_S_3_ interplay between structure, dielectric function, and amorphous-to-crystalline phase change for photonics

**DOI:** 10.1016/j.isci.2024.111206

**Published:** 2024-10-30

**Authors:** Yael Gutiérrez, Anna P. Ovvyan, Gonzalo Santos, Dilson Juan, Saul A. Rosales, Javier Junquera, Pablo García-Fernández, Stefano Dicorato, Maria M. Giangregorio, Elena Dilonardo, Fabio Palumbo, Mircea Modreanu, Josef Resl, Olga Ishchenko, Guy Garry, Tigers Jonuzi, Marin Georghe, Cornel Cobianu, Kurt Hingerl, Christoph Cobet, Fernando Moreno, Wolfram H.P. Pernice, Maria Losurdo

**Affiliations:** 1 Institute of Chemistry of Condensed Matter and Technologies for Energy, CNR ICMATE, Corso Stati Uniti 4, 35127, Padova, Italy; 2 Departmento de Fısica Aplicada, Universidad de Cantabria, Avda. Los Castros S/n, 39005 Santander, Spain; 3 Institute of Physics, University of Munster, Heisenbergstraße 11, 48149 Munster, Germany; 4 Departamento de Ciencias de La Tierra y Fısica de La Materia Condensada, Universidad de Cantabria, Cantabria Campus Internacional, Avda. de Los Castros S/n, 39005 Santander, Spain; 5 Institute of Nanotechnology, CNR-NANOTEC, Via Orabona 4, 70126 Bari, Italy; 6 Tyndall National Institute University College Cork, Lee Maltings, Dyke Parade, T12 R5CP Cork, Ireland; 7 Center for Surface and Nanoanalytics, Johannes Kepler University, 4040 Linz, Austria; 8 TE-OX, 21 Rue Jean Rostand, 91400 Orsay, France; 9 VLC Photonics S.L. Universidad Politecnica de Valencia (access I), Camino de Vera S/n, 46022 Valencia, Spain; 10 NANOM MEMS Srl, G. Cosbuc 9, 505400 Rasnov, Brasov, Romania; 11 Heidelberg University, Kirchhoff-Institute for Physics, Im Neuenheimer Feld 227, 69120 Heidelberg, Germany

## Main text

(iScience *25*, 104377; June 17, 2022)

In the originally published article, due to an error during production, affiliation 1 appeared as “CNR ICMATE, Corso Stati Uniti 4, I-35127 Padova, Italy.” The correct affiliation should be “Institute of Nanotechnology, CNR-NANOTEC, Via G. Amendola, 122, 70125 Bari, BA, Italy.”

