# Supplementary material for: Interlaboratory study on Sb2S3 interplay between structure, dielectric function, and amorphous-to-crystalline phase change for photonics
Source: iScience. 2022 May 10;25(6):104377. doi: 10.1016/j.isci.2022.104377 (PMC9127585; doi:10.1016/j.isci.2022.104377)

iScience, Volume *25*

Supplemental information

Interlaboratory study on Sb_2S_3 interplay between structure, dielectric function, and amorphous-to-crystalline phase change for photonics

Yael Gutiérrez, Anna P. Ovvyan, Gonzalo Santos, Dilson Juan, Saul A. Rosales, Javier Junquera, Pablo García-Fernández, Stefano Dicorato, Maria M. Giangregorio, Elena Dilonardo, Fabio Palumbo, Mircea Modreanu, Josef Resl, Olga Ishchenko, Guy Garry, Tigers Jonuzi, Marin Georghe, Cornel Cobianu, Kurt Hingerl, Christoph Cobet, Fernando Moreno, Wolfram H.P. Pernice, and Maria Losurdo

SUPPLEMENTAL INFORMATION

Figure S1. Example of a Mueller Matrix measured on amorphous Sb_2S_3 films. Related to Figure 6.

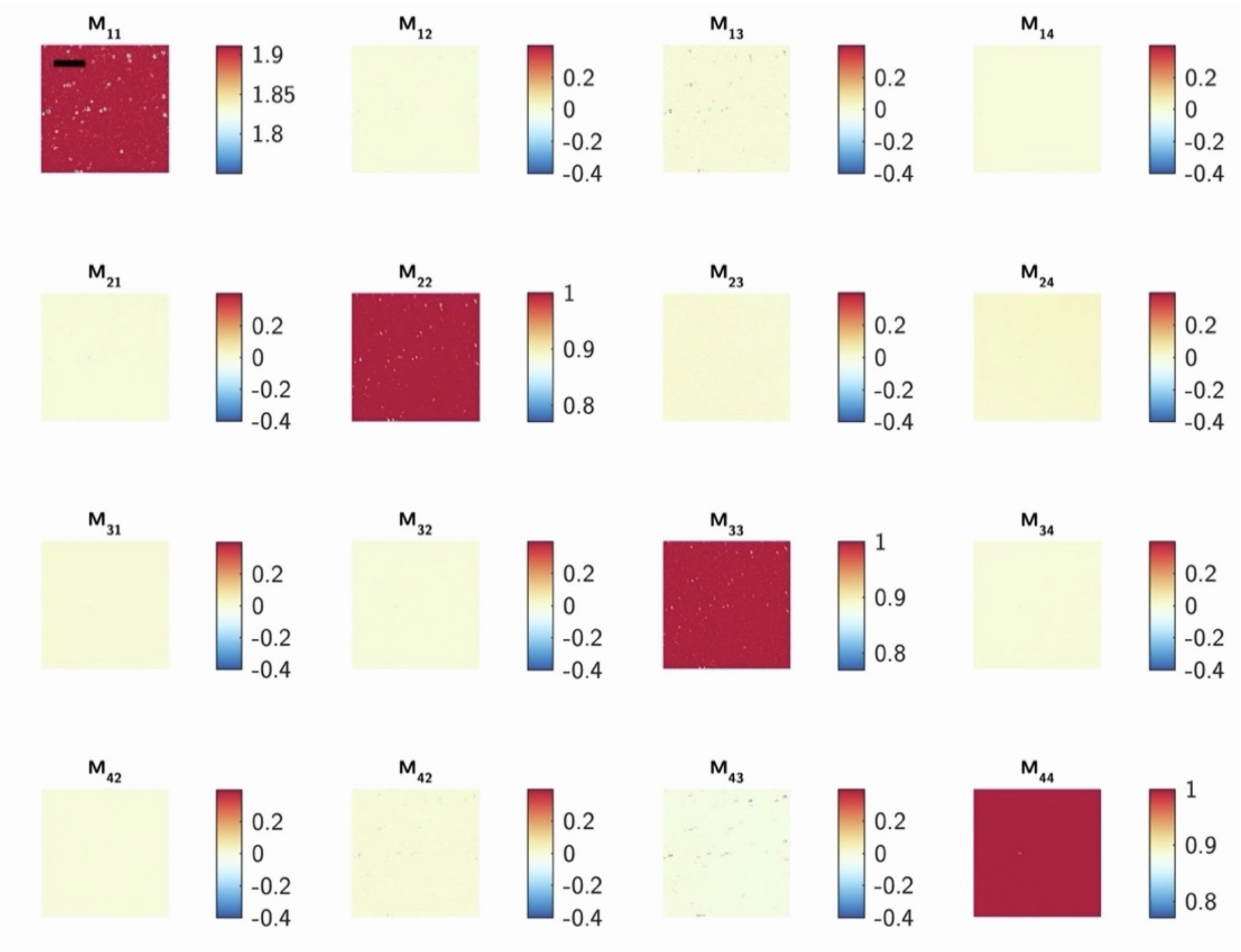

Figure S2. Mueller Matrix measured for *Type-I* crystallized Sb_2S_3 films thermally annealed at T = 250 – 300°C. Related to Figure 6a.

Sb_2S_3.

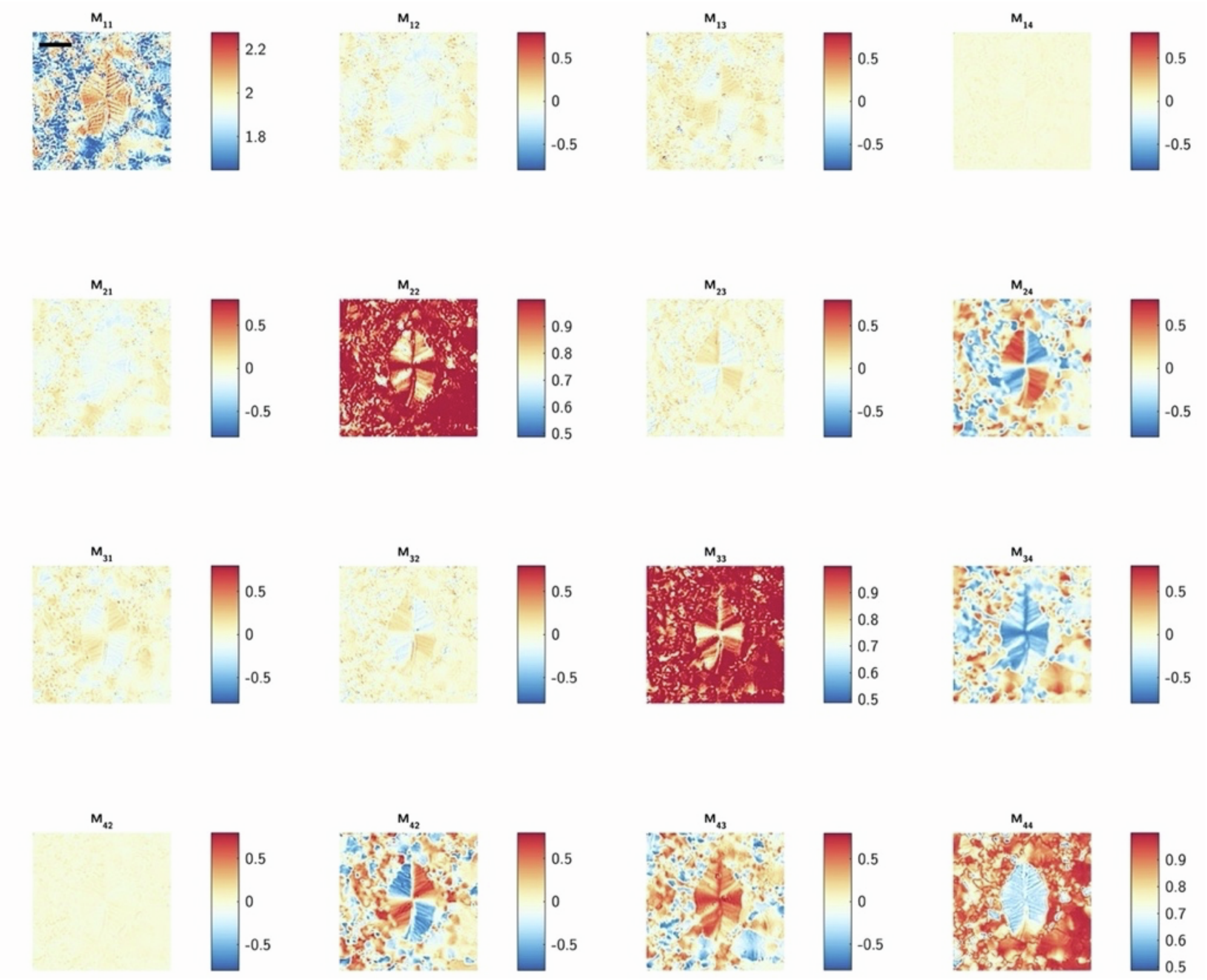

Figure S3. Mueller Matrix measured for *Type-II* crystallized Sb_2S_3 films thermally annealed at 350°C. Related to Figure 6b.

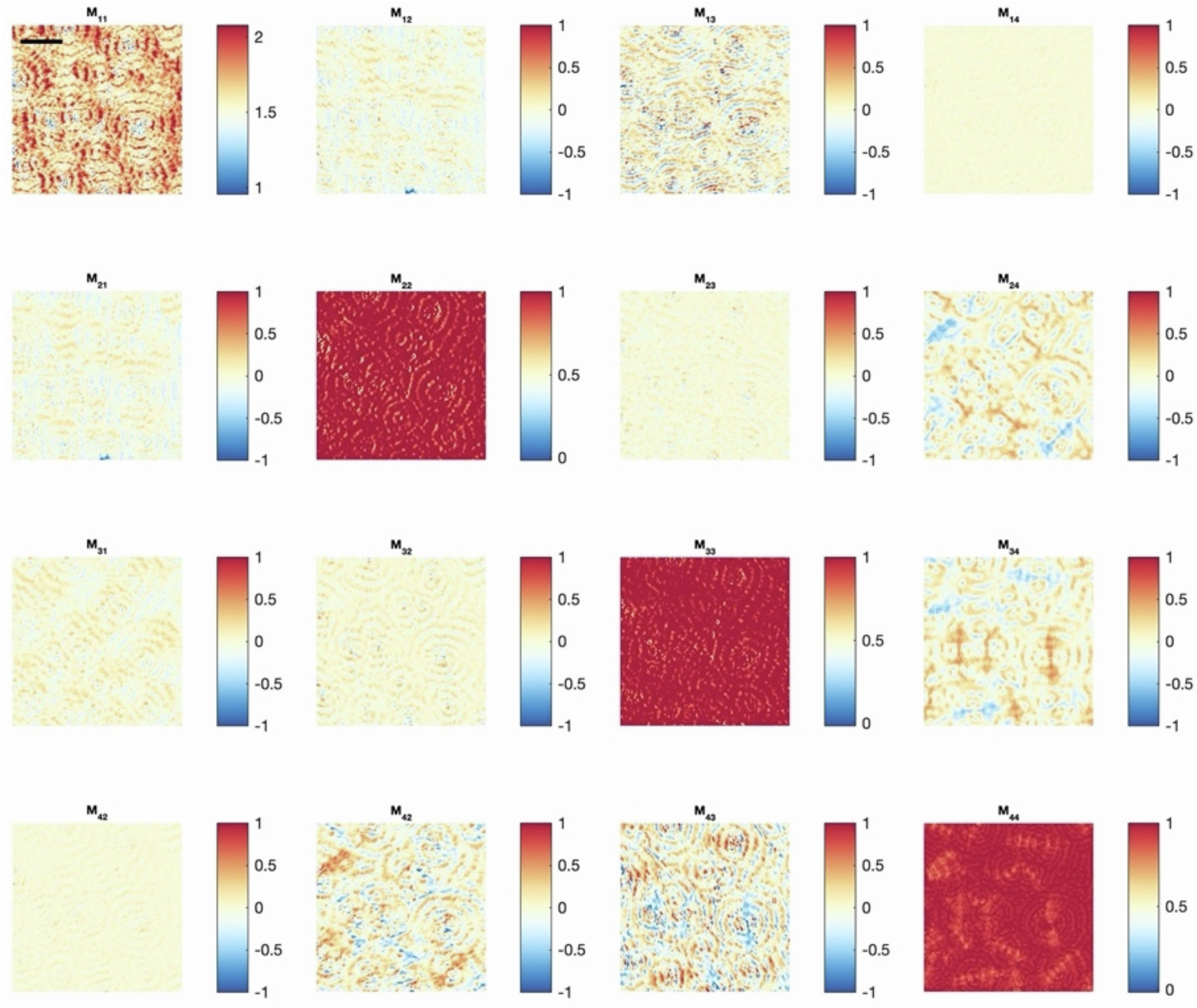

Figure S4. Crystal orientation in banded spherulitic crystals, Related to Figure 6b.

Sketch of the crystal orientation along the radial direction for the optical contrast in the periodic rings of the banded spherulites in *Type-II* crystalized Sb_2S_3 as seen in the micrograph.

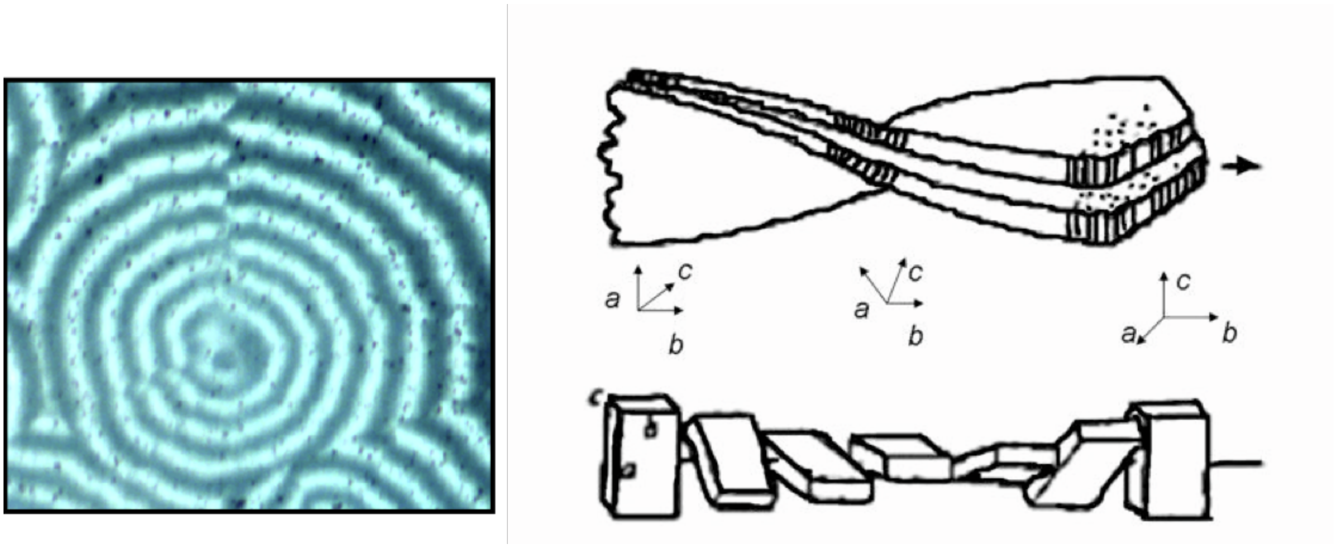

Supplement: Document S1. Figures S1–S4 [file mmc1.pdf]
